# Optimisation of Neuraminidase Expression for Use in Drug Discovery by Using HEK293-6E Cells

**DOI:** 10.3390/v13101893

**Published:** 2021-09-22

**Authors:** Ashley C. Campbell, John J. Tanner, Kurt L. Krause

**Affiliations:** 1 Department of Biochemistry, University of Otago, 710 Cumberland St., Dunedin 9016, New Zealand; ashley.campbell@otago.ac.nz; 2 Department of Biochemistry, University of Missouri, Columbia, MO 65211, USA; tannerjj@missouri.edu; 3 Department of Chemistry, University of Missouri, Columbia, MO 65211, USA; 4 Maurice Wilkins Centre for Molecular Biodiscovery, Auckland 1010, New Zealand

**Keywords:** neuraminidase, structural biology, eukaryotic protein expression, influenza, protein purification, transient transfection, chemokine binding protein

## Abstract

Influenza virus is a highly contagious virus that causes significant human mortality and morbidity annually. The most effective drugs for treating influenza are the neuraminidase inhibitors, but resistance to these inhibitors has emerged, and additional drug discovery research on neuraminidase and other targets is needed. Traditional methods of neuraminidase production from embryonated eggs are cumbersome, while insect cell derived protein is less reflective of neuraminidase produced during human infection. Herein we describe a method for producing neuraminidase from a human cell line, HEK293-6E, and demonstrate the method by producing the neuraminidase from the 1918 H1N1 pandemic influenza strain. This method produced high levels of soluble neuraminidase expression (>3000 EU/mL), was enhanced by including a secretion signal from a viral chemokine binding protein, and does not require co-expression of additional proteins. The neuraminidase produced was of sufficient quantity and purity to support high resolution crystal structure determination. The structure solved using this protein conformed to the previously reported structure. Notably the glycosylation at three asparagine residues was superior in quality to that from insect cell derived neuraminidase. This method of production of neuraminidase should prove useful in further studies, such as the characterisation of inhibitor binding.

## 1. Introduction

Influenza virus is an infectious, single-stranded RNA virus in the Orthomyxoviridae family that causes severe annual epidemics of respiratory disease resulting in high annual morbidity and mortality [1]. Further, influenza virus is capable of antigenic shift that may cause human pandemics, which can lead to much greater mortality [2]. Apart from vaccination, one of the most effective methods of treating or preventing influenza virus infection is the use of neuraminidase (NA) inhibitors (NAIs) [3,4,5]. NAIs bind at the active site of NA and attenuate the rate of enzymatic cleavage of sialic acid (SA). This cleavage event is required for influenza virus to exit host cells; therefore, infection of adjacent cells and subsequent viral transmission is prevented [6]. Persistent, but low background resistance to NAIs has been reported consistently [5,7] along with occasional hot spots of much higher resistance [7,8]. Given influenza virus’s ability to continually alter its genome to compensate for NAI use, it will be important to produce new generations of NAIs to complement the current antivirals [9].

The NA protein has been extensively characterized using structural biology, and its structure has played an important role in the development of NAIs [10,11,12,13,14,15,16,17]. Structure-aided studies, while very helpful, require the production of large amounts of NA, which can be challenging [16,18,19]. NA from influenza virus for X-ray crystallography has traditionally been produced in influenza virus infected embryonated hens eggs [18]. The hen’s egg method [18] is labour intensive, results in a low NA yield and can have safety and ethical concerns. In addition, the risks of propagating live virus are apparent, especially when investigating protein from more dangerous strains, such as the Spanish influenza virus strain A/BrevigMission/1/1918 (H1N1), which spread worldwide within months, infected around 30% of the global population and by some estimates led to 50 million fatalities [20].

To address the drawbacks of the hen’s egg method, recombinant NA production methods [19] have increasingly been explored using both prokaryotic and eukaryotic cell lines. Each of these methods has specific benefits and drawbacks. NA expression levels in prokaryotes are variable and have the important drawback of not being glycosylated as they would be in human infection [21]. Another major drawback involving NA produced in *Escherichia coli* is that the reducing environment of the bacterial cytosol is unsuitable for disulfide bond formation [22]. Expression in non-human eukaryotes has produced mixed results. For example, in yeast, the expressed NA has shown hyperglycosylation [21,23,24], resulting in a high molecular weight NA, which is not equivalent to the NA produced on the surface of infected human cells. NA expression in tobacco plants resulted in an insoluble product [25]. The use of baculovirus/insect cell expression systems has been more successful, resulting in large amounts of soluble and active NA [19,26], from which numerous X-ray crystal structures [26,27,28,29] and cryo-EM structures [30,31] have been solved. However, baculovirus systems can be challenging to master and maintain, and it can be argued that insect cells are less desirable than human cells to produce proteins arising from a human viral infection.

Recently, recombinant NA production was reported using human embryonic kidney (HEK) cells [32,33,34]. HEK cells are a human cell line transfected with adenovirus 5 DNA that has resulted in a permanent cell line capable of transient transfection [35]. The recombinant NA was found to be active, soluble, and glycosylated, indicating HEK expression is a suitable method for producing biologically relevant NA [32,36]. Using a transient transfection system in which expression in suspension HEK293-F cells was enhanced 4.2-fold through co-expression with NS1, Nivitchanyong et al. reported the production 41 EU/mL of NA from the HEK293-T cell expression line.

HEK293-E cells are a HEK cell line variant that is capable of growing in suspension and contains Epstein Barr Virus (EBV) nuclear antigen 1 (EBNA1), which allows them to use vectors containing an origin of replication from EBV [37]. Production levels of recombinant proteins from this suspension cell line have been reported as high as 50 mg/L in a transfected HEK293-E cell culture [37], with typical numbers ranging between 10 and 20 mg/L.

We report here the first use of HEK293-6E cells for the production of NA for structural studies, using H1N1 neuraminidase from the Spanish influenza virus strain A/BrevigMission/1/1918 (H1N1) as the test case. In addition, we have incorporated the wild type secretion signal from the CKBP protein from orf virus strain NZ2 (GenBank ABA00630.1) into our neuraminidase expression vector [38]. Our method allows for high-level NA expression in suspension culture (>3000 EU/mL) without the need for co-expression with the influenza virus NS1 protein. Purification by three chromatographic and two dialysis steps results in biologically relevant and natively folded NA. The recombinant NA is enzymatically active, glycosylated and crystallizes readily using commercial screens. The crystal structure of H1N1 was solved to 2.15 Å resolution and compared to a similar structure from baculovirus [26]. Our results suggest that HEK293-6E expression of NA has potential for use in biophysical studies aimed at the identification and characterisation of NAIs.

## 2. Materials and Methods

### 2.1. Cloning

Prior to NA expression trials, four genetic constructs were produced using modified Circular Polymerase Extension Cloning [39]. cDNA corresponding to residues 83 to 468 of the ectodomain of the NA from A/BrevigMission/1/1918 (18NA, EC:3.2.1.18) was cloned into the HEK293-6E expression vector pTT5. Prior to the start codon of the open reading frame a Kozac sequence (GCCACC) was included to provide a eukaryotic ribosomal binding site. To promote secretion of the expressed protein, a signal sequence (SS) from the chemokine binding protein (CKBP) from orf virus strain NZ2 (GenBank ABA00630.1), KAVLLLALLGAFTNA [38], was added at the N-terminus of NA, followed by a hexahistidine affinity tag (6 × His). Downstream of the 6 × His-tag, a 42 residue tetramerisation domain (TET) from human vasodilator-stimulated phosphoprotein was also included (SSSDYSDLQRVKQELLEEVKKELQKVKEEIIEAFVQELRKRG), to promote tetramerisation of the recombinant NA protein [26,40]. Three additional constructs were produced by deletion mutagenesis resulting in the following four forms, (+SS +TET), (+SS −TET), (−SS +TET), (−SS −TET), where a + sign indicates the sequence was included and a − sign indicates the sequence was absent from the construct.

### 2.2. Protein Expression

HEK293-6E cells were grown in suspension in Freestyle™ 293 expression Media (Gibco, Grand Island, NY, USA) supplemented with 1% Pluronic^®^ F-68 (Gibco, Grand Island, NY, USA) and Geneticin^®^ (Gibco, Grand Island, NY, USA). Cells were maintained at 37 °C, 120 r.p.m., in a sterile environment supplemented with 5% CO_2_. When the suspension cultures reached a concentration of 1 × 10^6^ cells/mL, the cells were transfected with the selected expression construct, using polyethyleneimine (PEI, Linear, MW 25000, Polysciences Inc, Warrington, PA, USA) as the transfection reagent. Small scale expression trials of NA were carried out by incubating cells for 6 days, at conditions described above, in a culture volume of 80 mL contained within a loosely capped 250 mL volumetric flask, to allow sufficient aeration. Initial test transfections were performed using a concentration of ~1 pg plasmid DNA and 3 pg of the transfection agent (PEI) per cell. Later, these amounts were optimised as described in Results. For large scale expression, 160 mL cultures were grown in 500 mL volumetric flasks. Typical large-scale expression was performed on a 1.6 L scale (10 separate 160 mL cultures).

### 2.3. Protein Purification

After removal of HEK cells by centrifugation at 6000× *g* for 20 min, concentrated (5×) His Trap binding buffer (100 mM Tris, 2.5 M NaCl, 100 mM imidazole, pH 7.4) was added to the supernatant to achieve a final concentration of 20 mM Tris, 500 mM NaCl, 20 mM imidazole, pH 7.4. It was very important to degas the supernatant was for at least 30 min, or significant foaming occurred in subsequent steps. The degassed supernatant was loaded onto a 5 mL His trap™ column (Fast Flow Crude, GE Healthcare, Uppsala, Sweden) equilibrated with binding buffer. After the loaded column was washed with binding buffer, proteins were eluted by applying a linear gradient of elution buffer (20 mM Tris, 500 mM NaCl, 500 mM imidazole, pH 7.4). The NA containing fractions were pooled and dialysed overnight against 4 L of 20 mM Tris buffer, pH 6.9, containing 150 mM KCl, 4 mM EDTA. The dialysis buffer also contained a separate dialysis bag containing ~20 g of activated charcoal. The next day, ammonium sulfate was slowly added to the dialysed sample containing NA, to achieve a final concentration of 1 M. This sample was then loaded onto a 1 mL Hi Trap Butyl HF hydrophobic interaction chromatography (HIC) column (GE), pre-equilibrated with HIC binding buffer (20 mM Na_2_HPO_4_, 1 M (NH_4_)_2_SO_4_, pH 7.4). Proteins were eluted with a 30 mL linear gradient of HIC elution buffer (20 mM Na_2_HPO_4_, pH 7.4) at 1 mL/min. Fractions containing pure NA were pooled and dialysed into SEC buffer (20 mM Tris, 200 mM NaCl, 2 mM CaCl_2_ pH 7.6) to remove excess (NH_4_)_2_SO_4_. The dialysed sample was then concentrated to 500 µL and loaded onto a Superose™ 12 size exclusion chromatography (SEC) column that had been equilibrated in SEC buffer (20 mM Tris, 200 mM NaCl, 2 mM CaCl_2_ pH 7.6). The central peak was collected and then concentrated ~20-fold to 8.5 mg/mL using a Vivaspin™ 500 (GE Healthcare, Buckinghamshire, UK).

### 2.4. Protein Analysis

Purified HEK-produced NA (HEK NA) was analysed by SDS-PAGE gel, mass spectrometry and enzyme assay. The SDS-PAGE gels were analysed optically for purity with estimates of purity made using band density analysis Image Lab™ software (BIO-RAD Laboratories, Hercules, California USA). Mass spectrometry was carried out on tryptic digests of proteins excised from SDS-PAGE gel bands which were analysed by the Centre for Protein Research at the University of Otago using liquid chromatography coupled nanospray LTQ-Orbitrap tandem mass spectrometry (LC-MS/MS). Protein concentration was determined using the Bradford assay [41].

### 2.5. Neuraminidase Assay

Neuraminidase assays were performed based on the procedures in Section 5.3 of WHO standard operating procedure 025—Fluorometric Neuraminidase Inhibition Assay (WHO, 2017) [42]. This assay measures the cleavage of 2′-(4-Methylumbelliferyl)-α-D-*N*-acetylneuraminic acid (MUNANA) to 4-methylumbelliferone (4-MU), catalysed by NA [43]. The assay is performed in an assay buffer containing 33.3 mM MES pH 6.0 and 4 mM CaCl_2_. To a black 96-well OptiPlate (Perkin Elmer, Roskilde, Denmark) 50 μL sample containing NA was added to a well. To start the assay 50 μL 300 μM MUNANA substrate was added, resulting in a final MUNANA concentration in the assay of 150 μM. Increasing 4-MU fluorescence was measured at excitation wavelength of 355 nm and an emission wavelength of 460 nm in a POLARstar Omega Plate Reader (BMG LABTECH, Ortenburg, Germany) for 20 min. To quantify the amount of cleaved MUNANA, a standard curve containing 4-MU at concentrations of 150, 75, 37.5, 18.75, 9.375, 4.6875, 2.34, 1.17, and 0 μM was included in the same volume as activity assays, and fluorescence measured at the same wavelengths. In this assay, 1 EU (enzyme unit) represents the amount of NA required to convert 1 µmol MUNANA to 4-MU per minute.

### 2.6. Glycosylation and Western Blot Analysis

Samples from small scale expression trials were separated by centrifugation (1000× *g*, for five minutes) into pellet (cells) and supernatant (expression media and secreted proteins) fractions. NA was found to express in the pellet in constructs lacking SS, i.e., NA (−SS − TET and − SS +TET) and in the supernatant for constructs with SS included NA (+SS − TET and +SS +TET).

Deglycosylation experiments on secreted NA were conducted on combined supernatants from days 3 to 6 following concentration by Vivaspin (10×). The Glycopro™ Enzymatic Deglycosylation Kit (Prozyme, Hayward, CA, USA) was used to remove sugars from the NA protein according to the manufacturer’s instructions. Deglycosylated samples were run on an SDS-PAGE gel (4–20% polyacrylamide) and transferred to PVDF membrane. The NA protein was detected using an anti-His primary antibody (Tetra-His antibody, mouse monoclonal IgG, Qiagen cat #34670, Hilden, Germany), and an anti-mouse IgG HRP-linked secondary antibody (Cell Signalling technology, Danvers, MA, USA). A parallel set of experiments were carried out in which NA was denatured prior to deglycosylation.

The protein sequence for NA from the A/BrevigMission/1/1918 strain was submitted to the NetNGlyc 1.0 Server for analysis [44]. The server analyses protein sequences for N-glycosylation sites and for a secretion signal using Signal P [45].

### 2.7. Crystallisation and Optimization

Crystallisation trials were performed at 4 and 18 °C using hanging drop vapour diffusion. A total of 192 crystallisation conditions were screened. The crystal screens tested were Wizard I and II random sparse matrix crystallization screens (Emerald BioSystems, Bainbridge Island, WA, USA), and Crystal Screen™ HR2-110 and HR2-112 (Hampton Research, CA, USA). Three hundred nanolitre drops (150 nL of 10 mg/mL NA in 20 mM Tris, 2 mM CaCl_2_, 150 mM NaCl, pH 8 and 150 nL precipitant) were prepared using a Mosquito^®^ Crystal robot (TTP Labtech) and incubated over a reservoir of 100 µL of the crystallization solution. A condition containing MES buffer and PEG 20,000 was selected for optimisation. Diffraction quality crystals were obtained by systematic variation of the pH in increments of 0.2 units, and the PEG 20,000 concentration in increments of 2%.

### 2.8. Data Collection and Processing

Harvested crystals were transferred into a solution containing 30% glycerol, 12% PEG 20,000, 100 mM MES, pH 6.5 for cryoprotection, and then flash-cooled in liquid nitrogen. X-ray data collection was carried out on the MX2 beamline equipped with an ADSC Quantum 315r Detector at the Australian Synchrotron (Melbourne, Australia) [46,47]. Data collection consisted of 360 frames at 0.5°/frame and 1 sec/frame collected at a distance of 300 mm with beam energy of 12,664.9 eV. XDS [48] and Aimless [49] were used for data processing. The NA crystallized in space group P2_1_ with unit cell parameters of a = 122.7 Å, b = 148.2 Å, c = 127.2 Å and β = 94.8°. Data collection and processing statistics are presented in Table 1.

### 2.9. Molecular Replacement, Model Building and Refinement

Molecular replacement was performed using MOLREP [50]. The 1918 NA structure (PDB ID 3BEQ, chain A), which was determined from protein produced in baculovirus infected insect cells and solved to 1.45 Å resolution, was used to generate a search model [26]. Prior to molecular replacement, chain B, water, ligands, and sugars were deleted. Analysis of the unit cell contents with the method of Matthews [51] had suggested 10 NA molecules in the asymmetric unit with 52% solvent and a V_M_ of 2.61 Å^3^/Da, but molecular replacement calculations indicated that the asymmetric unit contains eight monomers arranged as two tetramers. Thus, the actual solvent content is approximately 62%.

Refinement was performed using Phenix [52,53]. Model building was performed using Coot [54]. After side chains were manually fit to the density and optimized, water was then added using Phenix [52]. Waters with weak density or high B-factors were removed. Next, Ca^2+^ ions were added into strong electron density located in the known Ca^2+^ binding sites as reported previously [26]. Finally, sugars were added to the three defined N-glycosylation sites where clear density was observed using the Carbohydrate Module in Coot [55]. The structure was validated with MolProbity [56], PROCHECK [57], PDB CARE [58], CheckMyMetal [59] and the PDB validation server [60]. Table 1 lists the refinement statistics.

### 2.10. Structural Analysis

Structure figures were prepared using PyMOL (The PyMOL Molecular Graphics System, Version 1.8 Schrödinger, LLC) or Coot [54]. Analysis of tetrameric interfaces was performed using the PDBePISA server [61]. Structural analysis was also performed with PDBsum [62]. Electron Density for the A/BrevigMission/1/1918 neuraminidase structure, 3BEQ, was obtained from the EDS Server [63]. Polder maps [64] were generated with Phenix for glycosylated asparagine residues using the structure factors deposited into the PDB for both HEK NA and 3BEQ.

## 3. Results

### 3.1. Choice of Genetic Construct for NA Expression

**Figure 1:**
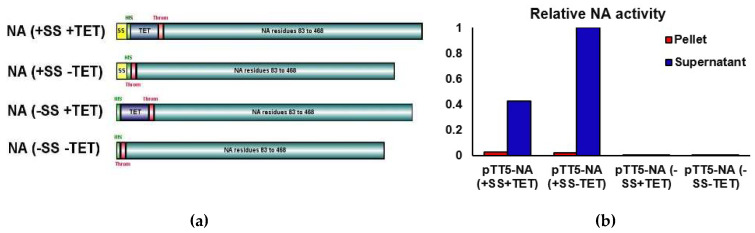

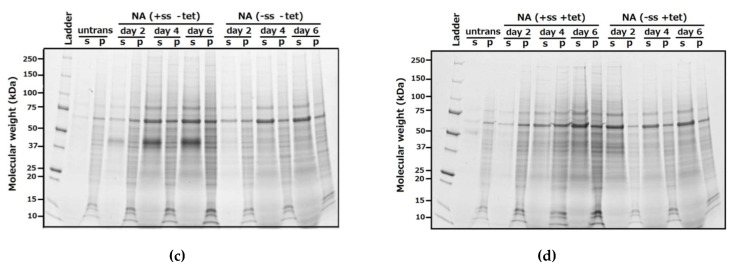
Optimisation of expression construct (**a**) H1N1 NA constructs used in this study. Each horizonal bar represents one of the four constructs used in this study. The secretion signal (SS) is shown in yellow, the His-tag is shown in green, the tetramerisation domain (TET) is shown in purple, the thrombin cleavage site is shown in red. This image was made with IBS software [65]. (**b**) Relative neuraminidase activity, plotted following normalization to the sample with the highest activity: pTT5-NA (+SS−TET). (**c**–**d**) SDS-Page gels (4–20%) from expression trials of NA (+SS +TET), (−SS +TET), (+SS −TET), (−SS −TET). The supernatant samples were concentrated 10× before loading. Untransfected cells (untrans) represent a negative control for NA expression. (**c**) shows expression of NA from constructs that did not contain a tetramerisation domain (−TET). (**d**) shows expression of NA from constructs which contained a tetramerisation domain (+TET). Both gels were stained with Coomassie Blue.

Four genetic constructs were produced for NA expression testing as described in Methods. All constructs contained residues 83 to 468 of the ectodomain of the NA from A/BrevigMission/1/1918 (18NA) along with an N-terminal His-tag. Two constructs contained a signal sequence (SS) from the chemokine binding protein (CKBP) from Orf virus (KAVLLLALLGAFTNA) [38] to promote extracellular secretion, and two constructs contained a 42 residue tetramerisation domain (TET) from human vasodilator-stimulated phosphoprotein to promote tetramerisation of the recombinant NA protein (Figure 1a) [26,40].

#### 3.1.1. Analysis of Expression by SDS-PAGE

To investigate the effects of the SS and TET domains on expression, the following four constructs were studied: (+SS +TET), (+SS −TET), (−SS +TET), (−SS −TET) (Figure 1). At 2, 4 and 6 days post transfection, cell culture supernatants (s) and cell pellets (p) were analysed by SDS-PAGE for NA expression. Of the four constructs, only the transfection with the plasmid pTT5-NA (+SS −TET) resulted in a prominent band on SDS-PAGE in the supernatant fractions located at ~45 kDa (Figure 1c). This molecular mass correlates with the predicted 44.5 kDa mass predicted for the NA construct and is consistent with the SS being cleaved upon secretion. This band was subsequently determined by mass spectrometry to be His-tagged NA from A/BrevigMission/1/1918 (data not shown). The intensity of the band on SDS-PAGE increased relative to untransfected cells over the six-day transfection period. Conversely, no obvious overexpression of NA using the other constructs (−SS −TET), (−SS +TET) and (+SS +TET), was observed on the SDS-PAGE gel at the predicted molecular weights of 44.5 or 49.5 kDa in either the supernatant or the pellet.

#### 3.1.2. NA Activity Assay

NA activity assays were performed to measure the relative level of NA activity in supernatant and lysed cell pellet samples following the 6-day transfection period. The highest relative NA activity was found in the supernatant of the NA (+SS −TET) transfection (Figure 1b), which was consistent with the SDS-PAGE analysis (Figure 1c). The supernatant activity was about 50 times that observed in the pellet for this construct suggesting that the secretion signal was effective. Interestingly the relative NA activity from the (+SS +TET) construct was consistent with a lower, but still significant, level of expression. These results indicate that the artificial tetramerisation domain is not necessary for the expression of the recombinant NA in this system. The NA (−SS −TET) and NA (−SS +TET) samples both had unmeasurable levels of NA activity, consistent with their absence of a band on the SDS-PAGE gels. This suggests the secretion signal is important for recombinant NA expression in HEK293-6E cells.

### 3.2. Optimisation of DNA Quantity and Up-Scaling for Larger Transfections

**Figure 2:**
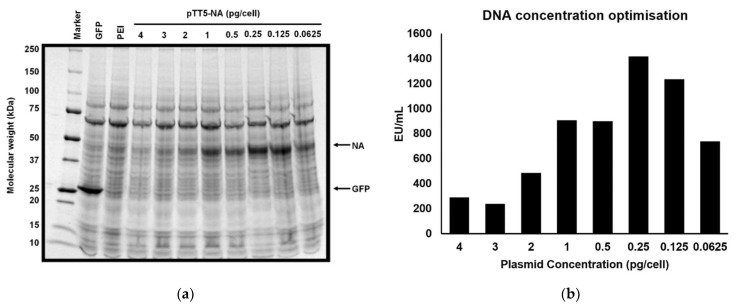
Optimisation of quantity of DNA in transfection for pTT5-NA (+SS −TET). 80 mL HEK293-6E cells (1 × 10^6^ cells/mL) were transfected with 4, 3, 2, 1, 0.5, 0.25, 0.125 or 0.0625 pg/cell pTT5-NA (+SS −TET) plasmid. Previous trials had been performed at 1 pg/cell. (**a**) 6 days post transfection cell culture supernatants were analysed by SDS-PAGE for NA expression. The supernatant samples were concentrated 10× before SDS-PAGE analysis. GFP represents a positive control for recombinant protein expression (27 kDa). PEI represents a negative control for recombinant protein expression. Samples were loaded onto SDS-PAGE gel (4–20%) and visualized using Coomassie blue. (**b**) Reducing the amount of plasmid DNA to 0.25 pg/cell increases NA production. Following transfection with varying amounts of pTT5-NA (+SS −TET), the cell supernatant was analysed after six days of expression to assess NA activity. DNA quantity per cell is shown on the x-axis. NA activity is plotted in EU/mL where 1 EU (enzyme unit) represents the amount of NA required to convert 1 µmol MUNANA to 4-MU per minute.

Based on the initial expression results, the plasmid pTT5-NA (+SS −TET) was selected for further optimisation and for larger scale NA production. Initial test transfections had been performed using a concentration of about 1 pg plasmid DNA per cell and 3 pg of the transfection agent (PEI) per cell. Given that altering the amount of DNA per cell can influence the amount of protein produced, [66,67,68] we analysed the amount of NA protein produced in the supernatant as a function of plasmid DNA per transfection. In Figure 2 the relative intensity of the NA band on the SDS-PAGE gel present at ~45 kDa increased as the DNA concentration was reduced from 4 pg/cell to 0.25 pg/cell (Figure 2a). However, further reduction of the DNA concentration to 0.125 pg/cell and to 0.0625 pg/cell, resulted in a diminished NA band. These results indicated that the optimal amount of DNA in the transfection is around 0.25 to 0.5 pg/cell. NA activity assays were performed on supernatant samples from the transfections (Figure 2b) and revealed that the highest level of NA activity, 1400 EU/mL, was observed in the transfection containing 0.25 pg/cell plasmid DNA, which is consistent with the results from the SDS-PAGE gel analysis. In larger scale transfections (1.6 L) NA expression as high as 3700 EU/mL was measured, although there was some variation between transfections.

### 3.3. Glycosylation Analysis

One potential advantage using HEK cells is that the recombinant NA produced is likely to have glycosylation modifications similar to NA produced during human infection. To analyse this, we studied the glycosylation of the NA produced during our transfections. Previous analysis of neuraminidase from the H1N1 pandemic strain suggests that it would have been glycosylated within the head domain of NA at Asn88, Asn146 and Asn234 [69]. These results were further corroborated following submission of the A/BrevigMission/1/1918 NA sequence to the NetNGlyc 1.0 Server [44].

**Figure 3:**
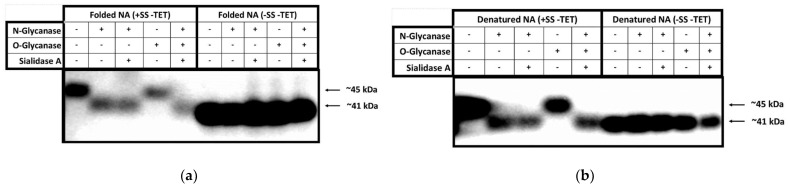
HEK NA deglycosylation analysed by Western blot. (**a**) The supernatant of a pTT5-NA (+SS −TET) and lysed pellet of pTT5-NA (−SS −TET) were digested with deglycosylation enzymes The digested proteins were separated by SDS-PAGE, transferred to PVDF membrane for analysis by Western blot. An anti-His antibody was utilised to visualize the NA proteins. (**b**) Proteins were denatured prior to deglycosylation, and then deglycosylated and analysed on WB in the same way as in panel a.

Deglycosylation experiments (Figure 3) were performed on supernatants obtained from the NA (+SS −TET) construct under native and denaturing conditions and then analysed to determine the extent and type of glycosylation (O- or N-linked), gain information about the accessibility of the glycosylation sites, and confirm cleavage of the secretion signal. In these deglycosylation experiments, NA (−SS −TET) was used as a negative control (Figure 3).

In the deglycosylation experiments conducted under native conditions, undigested NA (+SS −TET) migrates on SDS-PAGE at ~45 kDa. Following digestion with N-glycanase the size of this band is reduced to ~41 kDa, which is similar to the mobility of undigested NA (−SS −TET). This suggests that ~4 kDa of N-linked oligosaccharides have been cleaved from the surface of NA (+SS −TET). When digested with N-glycanase and Sialidase A, the same result occurs, which suggests that there are no α-2,8-linked terminal sialic acid moieties that hinder the glycanases accessibility to react with any of the N-linked sugar chains. When NA (+SS −TET) is digested with O-glycanase, the protein size remains ~45 kDa, indicating there are no O-linked glycosylations available on its surface. When digested with all deglycosylation enzymes the NA (+SS −TET) protein is ~41 kDa, the same as when digested with N-glycanase alone. As predicted, NA (−SS −TET) was unaffected by any of the deglycosylation enzymes, suggesting there were no glycosylation modifications on its surface. A slight difference in migration was noted between native NA (+SS −TET) protein, which had been deglycosylated, and NA (−SS −TET). The apparent larger molecular weight of deglycosylated NA (−SS −TET) could be interpreted as resulting from incomplete digestion by the deglycosylation enzymes.

The deglycosylation experiments using denatured NA protein show similar reductions in NA (+SS −TET) size for all digests, but more clearly demonstrates that the molecular mass of the N-glycanase processed NA is the same as NA (−SS −TET). This comparison suggests all glycosylation sites on NA (+SS −TET) are surface residues. The negative control, NA (−SS −TET), did not change in size following any of the digests because it is, as expected, an unglycosylated protein, due to the lack of a SS in the construct.

Finally, since it is clear from Figure 3 that the molecular weight of the band from denatured deglycosylated NA (+SS −TET) is the same as the undigested NA (−SS −TET), we can infer that NA (+SS −TET) has had its SS cleaved during HEK293-6E expression, otherwise it would result in a protein larger than NA (−SS −TET), but smaller than the glycosylated NA (+SS −TET). This result is consistent with mass spectrometry (data not shown).

### 3.4. Purification

**Figure 4:**
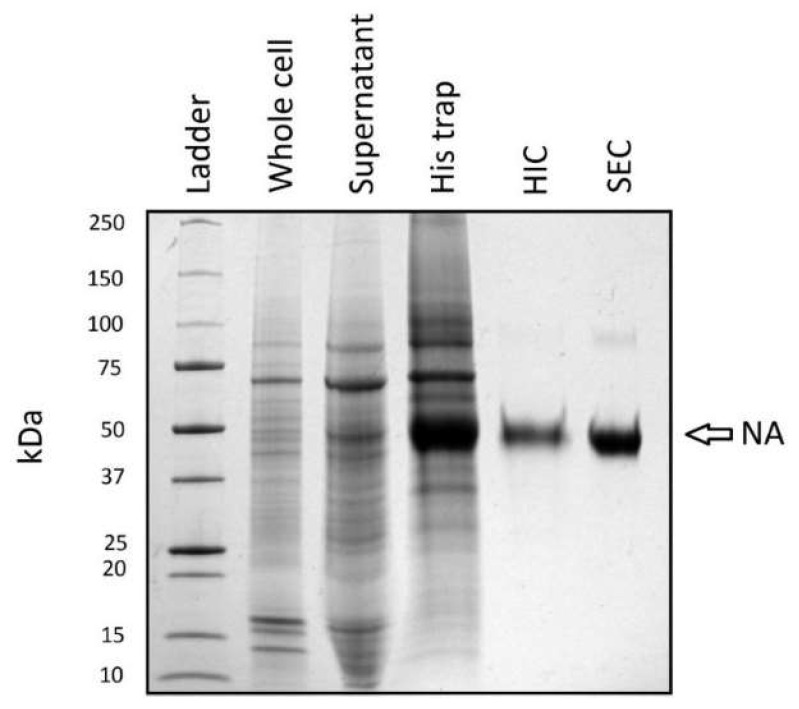
Purification of NA following HEK cell expression. SDS-PAGE (4–20%) separation of proteins obtained following key steps in NA purification. Lane 1: Mol Wt. standards. Lane 2: Whole cell lysate. Lane 3: 10× concentrated supernatant following expression. Lanes 4–6: His trap, HIC and SEC samples. Proteins were visualized using Coomassie Blue.

A purification strategy consisting of three chromatographic and two dialysis steps was performed following transient transfection of 1 L of HEK293-6E using the (+SS −TET) pTT5-NA construct. The purity of NA following each step can be followed by SDS-PAGE (Figure 4 and Table 2). The 45 kDa NA band is readily apparent in SDS-PAGE analysis of proteins in the supernatant following successful transfection. The subsequent metal affinity (IMAC) purification step results in a four-fold purification of NA (Table 2). Following this step, a major ~70 kDa contaminant was identified that proved difficult to separate from NA using typical chromatography. This persistent contaminant was determined to be Hsp70 by mass spectrometry (data not shown). To aid removal of Hsp70 by a subsequent chromatography step, the protein was dialyzed against 20 mM Tris, 150 mM KCl, 4 mM EDTA, pH 6.9 in the presence of activated charcoal that was sequestered in a separate dialysis bag (MWCO 12–14,000). This step proved crucial for eliminating Hsp70 co-migration with NA during hydrophobic interaction chromatography (HIC).

Following HIC, the NA band was visibly enriched (Figure 4) and 99% pure by gel densitometry analysis (Table 2). A final size exclusion chromatography (SEC) step was performed which resulted in a NA sample of >99% purity. A faint band migrating at ~90 kDa can be seen in Figure 4. Although the SDS-PAGE gel was performed under denaturing and reducing conditions, this band, as well as the 45 kDa band from the SEC lane, were confirmed as H1N1 (1918) NA by mass spectrometry (data not shown). The entire protocol achieved a 38.4-fold purification relative to the supernatant. The final yield is 1.4% of the NA present in the supernatant (Table 2) with significant losses recorded following the IMAC and HIC steps.

### 3.5. X-Ray Crystallography

#### 3.5.1. Crystallisation

**Figure 5:**
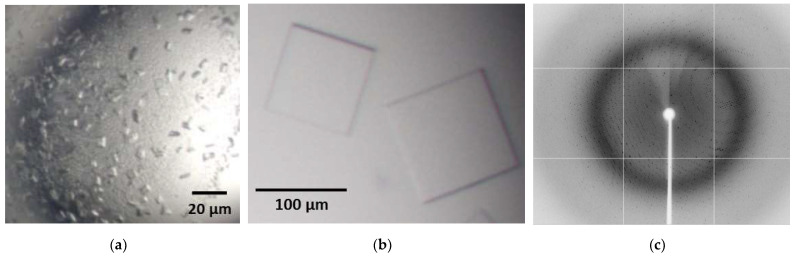
NA crystallization and X-ray diffraction. (**a**) Crystals in a hit condition containing 0.1 MES monohydrate pH 6.5, 12% *w/v* PEG-20,000. (**b**) Flat square plates (~100 µm × 100 µm × 5 µm) resulted from optimisation of hit condition. (**c**) X-ray diffraction image produced by produced by a H1N1 NA crystal collected at the MX2 beamline at the Melbourne Synchrotron. Maximum resolution of 2.04 Å at the edge of the detector, beam energy 12,664.9 eV, detector distance 300 mm, exposure time 1 s. A larger version of this diffraction image can be found in Figure S1.

Crystallisation experiments were setup at a NA concentration of 8.5 mg/mL using commercial screens as described in Methods. During a one-month observation period, crystals were observed in 27 out of the 192 conditions tested. The morphology of crystals ranged from clusters of thin needles to thin square plates. Crystals formed from conditions containing a range of precipitants including various PEG solutions (MW 400–20,000), glycerol, Jeffamine M-600^®^ (Hampton Research, Aliso Viejo, CA 92656, USA), ethylene glycol and ammonium sulfate. A range of buffers and pH values were found in wells with crystals including MES, Tris, HEPES, sodium citrate, potassium sodium tartrate, sodium acetate, sodium phosphate, CAPS and imidazole. The lowest pH solution in which crystals grew was pH 4.6 and the highest pH was 10.5. Following optimisation as described in methods, the crystallisation condition producing the best crystals contained 12% PEG 20,000, 100 mM MES, pH 6.5 with a drop ratio of 1 μL protein at 8.5 mg/mL to 1 μL reservoir. After four weeks of growth at 4 °C, crystals grown at this condition reached approximately 100 μm size (Figure 5).

#### 3.5.2. X-Ray Diffraction

Crystallographic data was collected on the MX2 beam line at the Australian Synchrotron (Melbourne, Australia) [47]. Diffraction from these NA crystals was well ordered and initially extended to 2.0 Å (Figure 5c and Figure S1) but radiation decay took place over the course of data collection, resulting in complete data being obtained to 2.15 Å. Data collection and refinement statistics are shown in Table 1.

### 3.6. Structural Comparison with Other NA Structures

#### 3.6.1. Overall Structure

**Figure 6:**
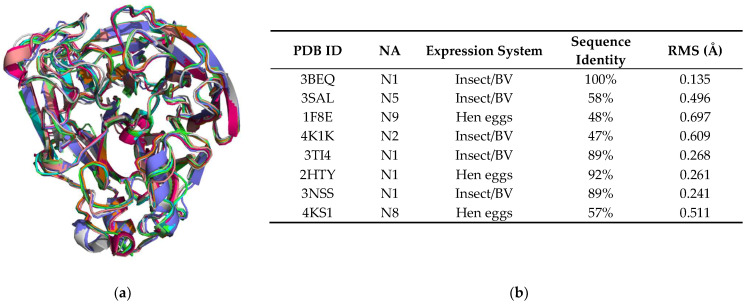
Overall structural comparison with other NA structures. (**a**) Structural alignment of NA monomers from various influenza virus strains and expression systems. Lime green: HEK NA, A/BrevigMission/1/1918 (H1N1). Cyan: 3BEQ, A/BrevigMission/1/1918 (H1N1). Light pink: 3SAL, A/duck/Alberta/60/1976 (H12N5). Grey: 1F8E, A/tern/Australia/G70C/1975 (H11N9). Purple: 4K1K, A/RI/5+/1957 (H2N2). Orange: 3TI4, A/California/04/2009 (H1N1). Dark Green: 2HTY, A/Vietnam/1203/04 (H5N1). Teal: 3NSS, A/California/04/2009 (H1N1). Hot pink: 4KS1, A/duck/Ukraine/1/1963 (H3N8). Chain A from each structure was chosen for the alignment. This superposition was performed in PyMOL, using the align command, which performs a sequence alignment, followed by a structural superposition. Water, metal ions and ligands were excluded from the alignment. A side-by-side comparison of HEK NA and 3BEQ can be seen in Supplemental Figure S2. (**b**) Differences between NA structures produced using HEK293-6E cells as compared to selected NA structures within the PDB. RMS differences were calculated in PyMOL, using the align command after exclusion of water, metal ions and ligands.

**Figure 7:**
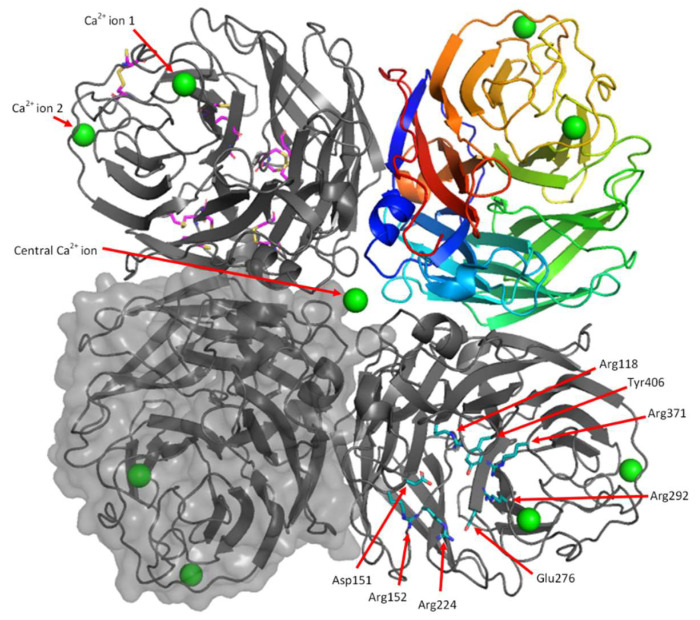
The H1N1 NA tetramer structure from HEK cell produced protein. The NA tetramer is made up of four monomers each related by a non-crystallographic four-fold axis of symmetry. The top left monomer is shown as a protein cartoon diagram in grey. The arrows represent β-strands and the spirals represent helical structure. Loops are shown as thin lines, connecting the β-strands and α-helices. The 8 disulfide bonds are shown as pink sticks. The top right monomer is shown as a protein cartoon diagram, with rainbow colouring from the N-terminus (blue) to C-terminus (red). The bottom left monomer is shown as a grey protein cartoon covered in a space filling molecular surface. The bottom right monomer is shown as grey cartoon, with the active site residues (Arg118, Asp151, Arg152, Arg224, Glu276, Arg292, Arg371, Tyr406) shown as blue sticks. The NA tetramer structure from HEK293-6E cell protein contains a total of 9 Ca^2+^ ions, which are shown as green spheres.

**Figure 8:**
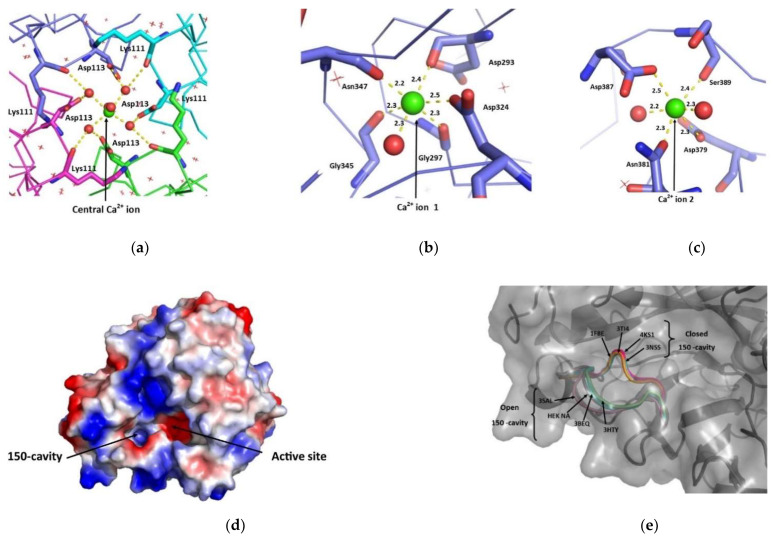
Conserved structural features of NA produced by HEK293-6E cells. (**a**) Coordination of the central Ca^2+^ ion. (**b**) Coordination of Ca^2+^ ion 1. (**c**) Coordination of Ca^2+^ ion 2. (**d**) Open 150-cavity observed for HEK NA. 150-cavities of other NAs can be found in supplemental Figure S4. (**e**) Position of 150s-loop. The HEK NA structure (chain A) is represented by a transparent grey surface and dark grey cartoon secondary structure. The 150s loop of published NA structures have been superposed onto the HEK NA structure. Cyan 3BEQ. Light pink 3SAL. Grey 1F8E. Purple 4K1K. Orange 3TI4. Dark Green 2HTY. Teal 3NSS. Hot pink 4KS1.

The structure of NA H1N1 produced from HEK293-6E cells has the expected 6-bladed β-propeller fold and contains the key elements found in all influenza virus NA structures [10,26,70,71] (Figure 6, Figure 7 and Figure 8). Each of the six blades is formed from four antiparallel β-strands, which are connected by loops. The H1N1 monomer structure aligns well with the 3BEQ structure reported earlier [26], as evidenced by the low RMS difference of 0.135 Å. Superposition of our HEK NA structure with eight representative NA structures from five classes (N1, N2, N5, N8 and N9) obtained from the PDB reveals an excellent fit with RMS differences ranging from 0.135 to 0.697 Å (Figure 6).

Each monomer is stabilised by seven or eight disulfide linkages which connect the following residues: Cys92-Cys417, Cys124-Cys129, Cys183-Cys230, Cys232-Cys237, Cys278-Cys291, Cys280-289, Cys421-447, Cys318-336 (Figure 7). The formation of these disulfide bonds in their native combinations suggests HEK cells have a suitable folding environment for NA. The disulfide bond between Cys318-Cys336 was broken in all chains, except chain E, possibly due to radiation damage in the X-ray beam. We also observed density for two conformations of other cysteine residues involved in disulfide bonds, so these were modelled in both the disulfide and free cysteine, with partial occupancies refined. The neuraminidase active site, which is comprised of Arg118, Asp151, Arg152, Arg224, Glu276, Arg292, Arg371 and Tyr406, is present in its expected conformation (Figure 7).

Structural analysis at each multimer interface shows twenty-two hydrogen bonds and one salt linkage are present [61], resulting in a total of eighty-eight hydrogen bonds and four salt linkages to form the tetramer. The hydrogen bonding network at the multimer interface is very similar to the dimer interface found in PDB file 3BEQ which contains the same bonding network when analysed by PISA [26].

The overall quality of the electron density is very good. The backbone of all residues is visible and almost all side chains could be modelled into the density. Exceptions to this included the side chains of Val83 (chain A), Lys150 (chains F and G), Lys259 (Chain A), Lys261 (Chain A) and Glu463 (Chains A, C, F–H). Weaker density was found at the C-terminus and 150s loop (Residues 148-151) in all chains and the 430s loop in chain H. These regions also had high B-factors (~60) compared to the average B-factor (26.8) of the structure. Chains D and H had somewhat higher overall B-factors. These two chains do not participate in major crystal contacts.

#### 3.6.2. Calcium Binding Sites

NA from H1N1 is known to bind two Ca^2+^ ions per monomer [26] and the HEK NA structure contains density that is consistent with this property. The Ca^2+^ ion at site 2 which is present in all eight NA monomers, has only been found, to date, in the 1918 H1N1 influenza virus NA structure (Figure 7 and Figure 8). The significance of this additional Ca^2+^ is not yet known.

Ca^2+^ ion 1 is coordinated by the backbone carbonyl groups of Asp293, Gly297, Gly345 and Asn347, the side chain of Asp324 and one water molecule (Figure 8b). Ca^2+^ ion 2 is coordinated by the side chains of Asp379, Asn381, Asp387, Ser389 and two water molecules (Figure 8c). The distances between atoms coordinating the Ca^2+^ are between 2.2 and 2.5 Å, which is within the range of 2.2–2.6 Å expected for Ca^2+^ - O interactions [72].

There is a third central calcium ion found in the centre of each tetramer which is, therefore, shared by all four monomers (Figure 7 and Figure 8a). This calcium has octahedral coordination by six water molecules, which in turn are coordinated by the backbone carbonyls of Lys111 and the side chains of Asp113 (Figure 8a). The coordination features of all calcium ions in the model are consistent with NA produced in baculovirus (PDB ID: 3BEQ) [26] and other proteins which bind Ca^2+^, and were successfully validated by CheckMyMetal [59].

#### 3.6.3. Active Site and 150-Cavity Structure

NA from different strains of influenza virus can be defined as belonging to Group 1 or Group 2, depending on its subtype. Within the Group 1 subtype a binding space has been identified (Figure 8d). This space, which is termed the 150-cavity, derives from movement of the 150s loop (Figure 8e) [65,73] and is a possible site for drug targeting. NA from A/BrevigMission/1/1918 (H1N1) is a Group 1 NA and in our structure the 150-cavity is predominantly found in the open form (Figure 8e). Main chain density was observed for the 150s loop in all monomers of the two tetramers, although it was challenging to fit some side chains (Figure S3). In seven of the eight chains, the 150s loop was found in the open conformation, as expected for ligand-free Group 1 NA. In contrast, for chain H the electron density in this region fits the structure in the closed conformation best, but has features consistent with partial occupancy for both the open and closed conformations. In all chains the 150s-loop had higher B-factors than the average for the structure. These higher B-factors are consistent with the results of molecular dynamics studies that suggest flexibility in this part of NA and migration between open and closed forms upon substrate binding [26,73,74].

Our results here are in good agreement with the insect cell derived NA structure [26] (3BEQ) which was reported as having an open 150-cavity. Since these two NA proteins were produced from differing expressions systems, this agreement is reassuring. Two other structures which show an open 150-cavity are 3SAL and 2HTY [73,75]. These represent an N5 and N1 NA, both belonging to the Group 1 NAs. Example structures with a closed 150s loop include 1F8E, 3TI4, 4KS1 or 3NSS, the 2009 H1N1 influenza virus, all Group 2 NAs [76,77,78,79]. Figure 8e includes illustrations of the 150s loop from the deposited structures of these NAs.

#### 3.6.4. Electron Density Evidence for Glycosylation

**Figure 9:**
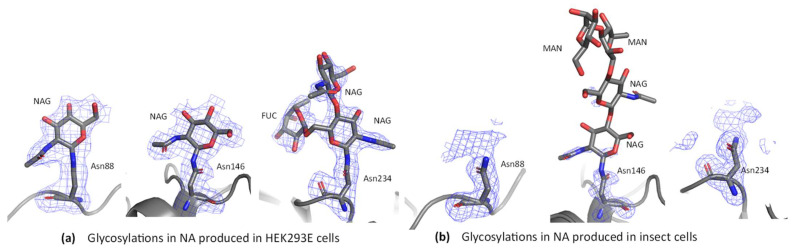
Glycosylations in NA. (**a**) NA glycosylations at Asn88, Asn146 and Asn234 in HEK293-6E produced NA. These images were made in PyMOL, with polder maps (blue mesh) contoured to 4σ. (**b**) For comparison the polder maps for the same residues from 3BEQ the insect cell H1N1 NA structure. Electron density for all chains can be found in Figures S5–S7.

Extra density was observed at the three predicted glycosylation sites asparagine residues 88, 146 and 234. At Asn88 and Asn146 one NAG residue could be built in all chains (Figure 9a, Figures S5a and S6a). At Asn234 two NAG residues and one fucose could be built into the density for several chains (Figure 9a and Figure S7a). The sugar geometry was validated with PDB CARE, which confirmed there were no issues with any of the sugars built into the structure [58]. As the average molecular weight of each sugar monomer is approximately 200 Da (mannose 180 Da, fucose 164 Da, N-acetylglucosamine 221 Da), we could have expected to observe as many as 20 sugar residues per NA chain, based on the molecular weight change of ~4 kDa described above in the deglycosylation experiment. Our result suggests that not all the sugars present on the surface of NA were observed, likely due to their inherent flexibility.

Comparison of glycosylation results obtained here with those of the 3BEQ structure may provide some insight into the effects of glycosylation in insect cells vs. HEK293-6E cells for NA. At Asn88 the HEK NA structure had density for one NAG residue, but no sugars could be built into the density at this site for 3BEQ (Figure 9b and Figure S5b). At Asn146 3BEQ had density that was characterized by the authors as “modest” and was modelled as NAG-NAG-MAN-MAN [26]. In HEK NA one NAG residue could be built in convincing density (Figure 9a and Figure S6b). Further, in the HEK NA structure, extra density consistent with the predicted O-sulfation site [80] at position 4 of NAG is present in chain A, but only NAG was built, as the density in the other chains was weak. At Asn234 two NAG residues and one fucose could be built into the density of the HEK NA, in five out of the eight chains (Figure 9a and Figure S6a). The remining chains had density for either one NAG and one fucose, or one NAG. In 3BEQ, no sugars were built at the Asn234 site (Figure 9b and Figure S6b). In summary, sugar residues at all predicted sites could be built in the HEK NA structure but not in 3BEQ. This difference in the structures suggests that human expression may offer an advantage regarding visualization of glycosylation, although we cannot rule out the possibility that differences in crystal packing also contribute to the stronger electron density for glycosylated residues in our structure.

## 4. Discussion

The global population remains largely susceptible to annual infections with the human influenza virus as well as to severe infections caused by pandemic strains [81]. Further, apart from vaccination, there are only two classes of drugs recommended for human influenza, NAIs (oseltamivir, peramivir and zanamivir) and the more recently approved polymerase acid endonuclease inhibitor, baloxavir [15,81,82]. The background resistance to treatment by currently approved NAIs is growing [9], and this indicates a need for more research on other possible drug targets of influenza virus, and a need to develop additional inhibitory drugs to current targets like NA [2,9]. Interestingly, combination therapy with oseltamivir and the recently approved PA endonuclease inhibitor baloxavir [83] has been shown to reduce the emergence of resistant influenza viruses in a mouse model [84].

In this work a simple, low labour-intensive expression strategy for NA production in HEK293-6E cells has been presented. This method offers the benefits of high-level expression of NA that is soluble, tetrameric, glycosylated, enzymatically active, and crystallises readily in a large range of conditions. The structure can be solved easily using phases determined by molecular replacement. This expression method may be useful for allowing researchers to replace labour intensive hens egg-based strategies which, in the case of pandemic avian strains, could require strict biosafety level 3 for protein isolation directly from live virus.

Compared with other examples of mammalian expression of NA [32], the method reported here using HEK293-6E cells produces a dramatically larger quantity of NA. Specifically, Nivitchanyong reported that NA activity of 41 EU/mL was reached on day 6 in their transfection supernatant [32], whereas here we report NA in the optimized transfection supernatant at 3700 EU/mL, which is nearly a 100-fold increase. Further, our results are scalable, and we were able to utilise a higher transfection volume of 1600 mL, with the total amount of NA produced being, therefore, about 6000-fold higher than the initial pilot HEK based trials [32]. However, comparisons of NA assays between labs must be interpreted with caution as results can vary when measuring fluorescence.

Our use of HEK293-6E cells for NA production can be compared to the use of HEK293-F and HEK293-T cell lines used by Nivitchanyong [32]. Growth in suspension has been mentioned as being a particularly valuable property of HEK293-6E as it allows for 3-dimentional accumulation of culture density, with more protein produced following transfection [37]. While HEK293-F cells like HEK293-E cells can be grown in suspension, the different cell lines employ different transfection vectors. Specifically, HEK293-6E cells have an EBV origin of replication and employ the pTT5 vector, while HEK293-F and HEK293-T use the pSecTag2/hygro A vector. Further, in the pTT5 construct used here, we incorporated an orf virus N-terminal chemokine binding protein secretion signal which proved to be important for greatly increased NA expression in the supernatant. To our knowledge this is the first example of a chemokine binding protein SS being used as an N-terminal tag to enhance expression and secretion of an unrelated protein, and may be applicable to other recombinant proteins expressed in HEK cells.

In Nivitchanyong et al., 2011 a murine Ig-κ chain leader secretion sequence, was employed [32]. In Nivitchanyong et al., 2011, NS1 co-expression was employed, and its inclusion resulted in a 4-fold improvement in yield. We also tested NS1 co-expression using HEK293-6E cells (data not shown) but in our hands it did not offer any improvements over the system reported here.

Apart from increased expression, the HEK NA protein produced from the HEK293-6E cells yielded an extremely pure NA sample (>99%) following purification which readily crystallized in a range of precipitants. Since finding suitable crystallisation conditions is often a rate limiting step in structure determination, the multitude of hits we obtained in crystallisation screens suggests this method will be useful for co-crystallisation with various inhibitors and antibodies.

One novel aspect of the purification reported here is its utilization of dialysis versus a solution containing EDTA and activated charcoal to remove co-migrating Hsp70 protein prior to HIC. Although successful in removing Hsp70, these steps did result in a large loss of NA protein. Overall, only 1.5% of the NA produced was able to be purified, which is a weakness of this method. In future work, the purification strategy may need to be further optimised to improve the yield.

The mechanism behind the success of this step could be that it allows for Hsp70 to be trapped in its open conformation in the following manner: EDTA chelates Mg^2+^ from the solution and the activated charcoal sequesters ATP/ADP. This prevents Hsp70 from hydrolysing ATP, and drives its equilibrium toward its open, unbound state. In this state NA is released and is, therefore, able to be purified by conventional methods [85]. However, more work would be needed to confirm this hypothesis.

Nevertheless, from only 1 L of culture multiple trays of crystallization experiments may be set up, saving the time and resources, compared with the large-scale egg-based expression systems used or large-scale insect cells. For instance Xu, et al. report their standard purification of NA from insect cells required a 9 L expression volume [26] in specialized CellSTACK (Corning) culture chambers as compared to the HEK293-6E method which can be performed on a 1 L scale in standard 250 mL Corning bottles or conical flasks.

Structural analysis of NA produced in HEK cells show good general agreement with NA produced using influenza virus infected hens eggs and NA produced using the BV system [26]. Little difference is seen in the tertiary and quaternary structure and comparisons of the tertiary structure show equivalent disulfide bond formation, calcium binding sites, active site conformation and 150s loop conformation.

Some differences between the HEK H1N1 NA structure and the insect cell produced H1N1 NA structure were found in the glycosylation sites. All three predicted sites in the HEK NA structure contain buildable sugar density versus only one in the BV structure. Notably, the Asn146 site in the BV structure contains more sugar moieties than the HEK derived site, but polder maps suggest this density may have been over modelled [64]. Another difference is the presence of fucose sugars found in the HEK NA structure at the Asn234 glycosite.

It is important to be able to visualise N-linked glycosylation of NA with as much accuracy as possible. Variations in glycosylation patterns have been shown have many roles in the influenza lifecycle. For example Östbye et al. reported that NA N-linked glycans were required for efficient viral incorporation and replication [86] and Bao et al. reported an importance of N-linked glycosylations for influenza virus budding [87]. The host range and zoonotic transmission of influenza virus is also affected by N-linked glycosylations of NA [88]. It is advantageous to be able to visualize fucose as it has been implicated in both the tetramerisation and activity of NA purified from native sources [89]. It has also been proposed that the abundance of fucose found in NA glycosites may be a determinant for virulence of influenza virus [80]. Since it may contribute to the antibody-NA interaction interface, having buildable fucose in the structure could be helpful for structural studies of antibody-NA interactions during vaccine development.

The production of tetrameric NA is another advantage of the HEK293-6E expression system. Viral NA requires tetramerisation for biologic activity [90]. It has been shown using molecular dynamic simulations that tetramerisation of NA subtly changes the structural geometry of the NA and contributes to stabilisation of the calcium binding sites, active site, and 150s loop [91], suggesting a rigid monomeric structure cannot simply be replicated and transposed to represent the true biological tetramer. The steps in tetramerisation of native NA have be shown to involve initial tertiary folding in the ER (which is reliant on intramolecular disulfide bond formation), monomers binding to form dimers via an intermolecular disulfide linkage in the stalk, conformational change, and finally tetramerisation [92]. This final step is dependent on glycosylation, and incorrect glycosylation can result in only the dimeric form of NA being produced [92].

Since NA tetramer formation is likely to be dependent on glycosylation events that can take place within human cell lines [92], a major benefit of the HEK based system for producing NA is that the tetrameric form of NA was obtained in the crystal structure, and in solution based on DLS measurements (data not shown). It is interesting that we found that the HEK293-6E system produces tetrameric NA even though our constructs did not contain the stalk region with its disulfide linkages. We also found that a tetramerisation domain within our expression construct was not needed as our +SS −TET construct readily yielded tetramers.

The approach we have described to viral protein production could readily be applied to other viral proteins. It would be most likely to work for proteins that are known to be secreted and soluble in extracellular media. The protein sequence would be incorporated into the pTT5 vector as described by Durocher [37] but with the addition of including the secretion tag from orf virus CKBP. Of course, other secretion tags could be trialled as well including the wild-type tag if the protein is normally secreted, but in our hands the secretion signal from orf virus CKBP is very potent.

Future applications of this work include attempts to conduct inhibitor binding studies and the structural analysis of antibody-NA interactions. In addition, all current NA X-ray structures contain only the head domain of NA and omit the stalk and transmembrane domain (residues 1–82). Since HEK cell lines have potential as suitable producers of crystallisable membrane proteins [93,94,95], this expression system may permit the expression and correct folding of the full-length NA. With careful optimisation, perhaps full-length NA could be purified and crystallised, with the goal of observing the molecular details of the stalk and transmembrane region. Insight to the structure of the NA stalk and transmembrane domain would be useful, as a shortened stalk region has been shown to increase influenza virus H5N1 virulence in avian wildlife [96,97], yet the structural mechanism for this remains elusive. Abundant expression of soluble and crystallisable NA from the secretion optimised HEK293-6E based system can help accelerate research on this important biomedical target.

## Figures and Tables

**Table 1:** Data collection and refinement statistics ^a^.

Data Collection	
Space group	P 2_1_
Cell dimensions	
a, b, c (Å)	122.7, 148.2, 127.2
α, β, γ (°)	90.0, 94.8, 90.0
Wavelength (Å)	0.9790
Resolution range (Å)	2.15–48.15 (2.15–2.19)
Observations	914,067 (45,188)
Unique reflections	242,924 (11,945)
*R*_merge_ (*I*)	0.241 (1.183)
*R*_meas_(*I*)	0.281 (1.38)
*R*_pim_ (*I*)	0.144 (0.703)
Mean I/σ	6.1 (1.5)
Mean *CC*_1/2_	0.977 (0.412)
Completeness (%)	99.1 (98.4)
Multiplicity	3.8 (3.8)
No. of atoms	25,641
Protein	23,774
Carbohydrate	476
Ca^2+^	18
Glycerol	36
Water	1345
R_work_	0.1858
R_free_^b^	0.2151
RMSD from ideal	
Bond length (Å)	0.009
Bond angle (°)	0.970
Ramachandran Plot	
Favoured (%)	95.3
Outliers (%)	0.07
Clash score (%-tile) ^c^	100
MolProbity score (%-tile) ^c^	100
B-factors (Å^2^)	
Average	26.8
Protein	25.8
Carbohydrate	65.2
Ca^2+^	29.8
Glycerol	45.5
Water	21.1
Coordinate error (Å) ^d^	0.27
PDB ID	6D96

^a^ Values for the outer resolution shell of data are given in parenthesis. ^b^ 3% test set. ^c^ From MolProbity. ^d^ Maximum likelihood-based coordinate error estimate reported by phenix.refine.

**Table 2:** NA Purification.

Step	Volume (mL)	Purity (BIORAD)	[Protein] (μg/mL)	Total Protein (μg)	Total NA (μg)	Yield (%) ^a^	NA Activity (EU/mL)	Total EU	Specific Activity (EU/μg) ^b^	Fold Purification ^c^
Supernatant	1000	14	199	198,700	27,818	100	3.7 × 10^3^	3.7 × 10^6^	18	1
His-Trap	66	47	260	17,173	8071	29	1.9 × 10^4^	1.2 × 10^6^	75	4
HIC	9	100	80	727	727	2.6	2.7 × 10^4^	2.4 × 10^5^	336	18
SEC	1.5	100	269	403	403	1.4	1.9 × 10^5^	2.8 × 10^5^	710	38

^a^ Yield was calculated using the final total NA activity divided by the initial total NA found in the supernatant prior to purification. ^b^ The specific activity was calculated by dividing total EU by total protein. ^c^ Fold purification was calculated by dividing the specific activity by the specific activity of the supernatant.

## Data Availability

Coordinates and structure factor amplitudes have been deposited in the Protein Data Bank under accession code 6D96.

## Supplementary Materials

The following are available online at https://www.mdpi.com/article/10.3390/v13101893/s1, Figure S1: Enlarged X-ray diffraction image from an H1N1 NA crystal, Figure S2: Side-by-side comparison of HEK NA and 3BEQ. Figure S3: 150-cavity of the 8 chains in HEK NA, Figure S4: Active site and 150-cavity of selected NA structures, Figure S5: NA glycosylations at Asn88, Figure S6: NA glycosylations at Asn146, Figure S7: NA glycosylations at Asn234.

## References

[1] Pleschka S. (2013). Overview of influenza viruses. Curr. Top. Microbiol. Immunol..

[2] Crusat M., de Jong M.D. (2007). Neuraminidase inhibitors and their role in avian and pandemic influenza. Antivir. Ther..

[3] Tejada S., Jansson M., Solé-Lleonart C., Rello J. (2021). Neuraminidase inhibitors are effective and safe in reducing influenza complications: Meta-analysis of randomized controlled trials. Eur. J. Intern. Med..

[4] Hayden F.G., Atmar R.L., Schilling M., Johnson C., Poretz D., Paar D., Huson L., Ward P., Mills R.G. (1999). Use of the selective oral neuraminidase inhibitor oseltamivir to prevent influenza. N. Engl. J. Med..

[5] Takashita E., Daniels R.S., Fujisaki S., Gregory V., Gubareva L.V., Huang W., Hurt A.C., Lackenby A., Nguyen H.T., Pereyaslov D. (2020). Global update on the susceptibilities of human influenza viruses to neuraminidase inhibitors and the cap-dependent endonuclease inhibitor baloxavir, 2017–2018. Antivir. Res..

[6] Santesso N., Hsu J., Mustafa R., Brozek J., Chen Y.L., Hopkins J.P., Cheung A., Hovhannisyan G., Ivanova L., Flottorp S.A. (2013). Antivirals for influenza: A summary of a systematic review and meta-analysis of observational studies. Influenza Other Respir. Viruses.

[7] Lee N., Hurt A.C. (2018). Neuraminidase inhibitor resistance in influenza: A clinical perspective. Curr. Opin. Infect. Dis..

[8] McKimm-Breschkin J.L., Selleck P.W., Usman T.B., Johnson M.A. (2007). Reduced sensitivity of influenza A (H5N1) to oseltamivir. Emerg. Infect. Dis..

[9] McKimm-Breschkin J.L. (2013). Influenza neuraminidase inhibitors: Antiviral action and mechanisms of resistance. Influenza Other Respir. Viruses.

[10] Colman P.M., Varghese J.N., Laver W.G. (1983). Structure of the catalytic and antigenic sites in influenza virus neuraminidase. Nature.

[11] Baker A.T., Varghese J.N., Laver W.G., Air G.M., Colman P.M. (1987). Three-dimensional structure of neuraminidase of subtype N9 from an avian influenza virus. Proteins.

[12] Von Itzstein M., Wu W.-Y., Kok G.B., Pegg M.S., Dyason J.C., Jin B., Phan T.V., Smythe M.L., White H.F., Oliver S.W. (1993). Rational design of potent sialidase-based inhibitors of influenza virus replication. Nature.

[13] Colman P.M. (1994). Influenza virus neuraminidase: Structure, antibodies, and inhibitors. Protein Sci. A Publ. Protein Soc..

[14] McKimm-Breschkin J.L., Sahasrabudhe A., Blick T.J., McDonald M., Colman P.M., Hart G.J., Bethell R.C., Varghese J.N. (1998). Mutations in a conserved residue in the influenza virus neuraminidase active site decreases sensitivity to Neu5Ac2en-derived inhibitors. J. Virol..

[15] Colman P.M. (2002). Neuraminidase inhibitors as antivirals. Vaccine.

[16] Smith B.J., McKimm-Breshkin J.L., McDonald M., Fernley R.T., Varghese J.N., Colman P.M. (2002). Structural studies of the resistance of influenza virus neuramindase to inhibitors. J. Med. Chem..

[17] Von Itzstein M., Thomson R. (2009). Anti-influenza drugs: The development of sialidase inhibitors. Handb. Exp. Pharmacol..

[18] McKimm-Breschkin J.L., Caldwell J.B., Guthrie R.E., Kortt A.A. (1991). A new method for the purification of the influenza A virus neuraminidase. J. Virol. Methods.

[19] Schmidt P.M., Attwood R.M., Mohr P.G., Barrett S.A., McKimm-Breschkin J.L. (2011). A generic system for the expression and purification of soluble and stable influenza neuraminidase. PLoS ONE.

[20] Taubenberger J.K., Reid A.H., Fanning T.G. (2000). The 1918 influenza virus: A killer comes into view. Virology.

[21] Yang Y.L., Chang S.H., Gong X., Wu J., Liu B. (2012). Expression, purification and characterization of low-glycosylation influenza neuraminidase in alpha-1,6-mannosyltransferase defective Pichia pastoris. Mol. Biol. Rep..

[22] Ma Y., Lee C.J., Park J.S. (2020). Strategies for Optimizing the Production of Proteins and Peptides with Multiple Disulfide Bonds. Antibiotics.

[23] Martinet W., Saelens X., Deroo T., Neirynck S., Contreras R., Min Jou W., Fiers W. (1997). Protection of mice against a lethal influenza challenge by immunization with yeast-derived recombinant influenza neuraminidase. Eur. J. Biochem..

[24] Shigemori T., Nagayama M., Yamada J., Miura N., Yongkiettrakul S., Kuroda K., Katsuragi T., Ueda M. (2013). Construction of a convenient system for easily screening inhibitors of mutated influenza virus neuraminidases. FEBS Open Bio.

[25] Pua T.L., Loh H.S., Massawe F., Tan C.S., Omar A.R. (2012). Expression of Insoluble Influenza Neuraminidase Type 1 (NA1) Protein in Tobacco. J. Trop. Life Sci..

[26] Xu X., Zhu X., Dwek R.A., Stevens J., Wilson I.A. (2008). Structural characterization of the 1918 influenza virus H1N1 neuraminidase. J. Virol..

[27] Streltsov V.A., Schmidt P.M., McKimm-Breschkin J.L. (2019). Structure of an Influenza A virus N9 neuraminidase with a tetrabrachion-domain stalk. Acta Cryst. F Struct. Biol. Commun..

[28] Wan H., Gao J., Yang H., Yang S., Harvey R., Chen Y.-Q., Zheng N.-Y., Chang J., Carney P.J., Li X. (2019). The neuraminidase of A(H3N2) influenza viruses circulating since 2016 is antigenically distinct from the A/Hong Kong/4801/2014 vaccine strain. Nat. Microbiol..

[29] Yang H., Carney P.J., Mishin V.P., Guo Z., Chang J.C., Wentworth D.E., Gubareva L.V., Stevens J. (2016). Molecular Characterizations of Surface Proteins Hemagglutinin and Neuraminidase from Recent H5Nx Avian Influenza Viruses. J. Virol..

[30] Zhu X., Turner H.L., Lang S., McBride R., Bangaru S., Gilchuk I.M., Yu W., Paulson J.C., Crowe J.E., Ward A.B. (2019). Structural Basis of Protection against H7N9 Influenza Virus by Human Anti-N9 Neuraminidase Antibodies. Cell Host Microbe.

[31] Madsen A., Dai Y.N., McMahon M., Schmitz A.J., Turner J.S., Tan J., Lei T., Alsoussi W.B., Strohmeier S., Amor M. (2020). Human Antibodies Targeting Influenza B Virus Neuraminidase Active Site Are Broadly Protective. Immunity.

[32] Nivitchanyong T., Yongkiettrakul S., Kramyu J., Pannengpetch S., Wanasen N. (2011). Enhanced expression of secretable influenza virus neuraminidase in suspension mammalian cells by influenza virus nonstructural protein 1. J. Virol. Methods.

[33] Ecker J.W., Kirchenbaum G.A., Pierce S.R., Skarlupka A.L., Abreu R.B., Cooper R.E., Taylor-Mulneix D., Ross T.M., Sautto G.A. (2020). High-Yield Expression and Purification of Recombinant Influenza Virus Proteins from Stably-Transfected Mammalian Cell Lines. Vaccines.

[34] Van der Woude R., Turner H.L., Tomris I., Bouwman K.M., Ward A.B., de Vries R.P. (2020). Drivers of recombinant soluble influenza A virus hemagglutinin and neuraminidase expression in mammalian cells. Protein Sci. A Publ. Protein Soc..

[35] Thomas P., Smart T.G. (2005). HEK293 cell line: A vehicle for the expression of recombinant proteins. J. Pharmacol. Toxicol. Methods.

[36] Kongkamnerd J., Milani A., Cattoli G., Terregino C., Capua I., Beneduce L., Gallotta A., Pengo P., Fassina G., Monthakantirat O. (2011). The quenching effect of flavonoids on 4-methylumbelliferone, a potential pitfall in fluorimetric neuraminidase inhibition assays. J. Biomol. Screen..

[37] Durocher Y., Perret S., Kamen A. (2002). High-level and high-throughput recombinant protein production by transient transfection of suspension-growing human 293-EBNA1 cells. Nucleic Acids Res..

[38] Couñago R.M., Fleming S.B., Mercer A.A., Krause K.L. (2010). Crystallization and preliminary X-ray analysis of the chemokine-binding protein from orf virus (Poxviridae). Acta Cryst. Sect. F Struct. Biol. Cryst. Commun..

[39] Quan J., Tian J. (2011). Circular polymerase extension cloning for high-throughput cloning of complex and combinatorial DNA libraries. Nat. Protoc..

[40] Kuhnel K., Jarchau T., Wolf E., Schlichting I., Walter U., Wittinghofer A., Strelkov S.V. (2004). The VASP tetramerization domain is a right-handed coiled coil based on a 15-residue repeat. Proc. Natl. Acad. Sci. USA.

[41] Bradford M.M. (1976). A rapid and sensitive method for the quantitation of microgram quantities of protein utilizing the principle of protein-dye binding. Anal. Biochem..

[42] Leang S.-K., Hurt A.C. (2017). Fluorescence-based Neuraminidase Inhibition Assay to Assess the Susceptibility of Influenza Viruses to The Neuraminidase Inhibitor Class of Antivirals. J. Vis. Exp..

[43] Potier M., Mameli L., Belisle M., Dallaire L., Melancon S.B. (1979). Fluorometric assay of neuraminidase with a sodium (4-methylumbelliferyl-alpha-d-*N*-acetylneuraminate) substrate. Anal. Biochem..

[44] Gupta R., Brunak S. (2002). Prediction of glycosylation across the human proteome and the correlation to protein function. Pac. Symp. Biocomput..

[45] Nielsen H. (2017). Predicting Secretory Proteins with SignalP. Methods Mol. Biol..

[46] McPhillips T.M., McPhillips S.E., Chiu H.J., Cohen A.E., Deacon A.M., Ellis P.J., Garman E., Gonzalez A., Sauter N.K., Phizackerley R.P. (2002). Blu-Ice and the Distributed Control System: Software for data acquisition and instrument control at macromolecular crystallography beamlines. J. Synchrotron Radiat..

[47] Aragão D., Aishima J., Cherukuvada H., Clarken R., Clift M., Cowieson N.P., Ericsson D.J., Gee C.L., Macedo S., Mudie N. (2018). MX2: A high-flux undulator microfocus beamline serving both the chemical and macromolecular crystallography communities at the Australian Synchrotron. J. Synchrotron Radiat..

[48] Kabsch W. (2010). Integration, scaling, space-group assignment and post-refinement. Acta Crystallogr. Sect. D Biol. Crystallogr..

[49] Evans P.R., Murshudov G.N. (2013). How good are my data and what is the resolution?. Acta Crystallogr. Sect. D Biol. Crystallogr..

[50] Vagin A., Teplyakov A. (2010). Molecular replacement with MOLREP. Acta Crystallogr. Sect. D Biol. Crystallogr..

[51] Matthews B.W. (1968). Solvent content of protein crystals. J. Mol. Biol..

[52] Adams P.D., Afonine P.V., Bunkoczi G., Chen V.B., Davis I.W., Echols N., Headd J.J., Hung L.-W., Kapral G.J., Grosse-Kunstleve R.W. (2010). PHENIX: A comprehensive Python-based system for macromolecular structure solution. Acta Crystallogr. Sect. D.

[53] McCoy A.J., Grosse-Kunstleve R.W., Adams P.D., Winn M.D., Storoni L.C., Read R.J. (2007). Phaser crystallographic software. J. Appl. Cryst..

[54] Emsley P., Cowtan K. (2004). Coot: Model-building tools for molecular graphics. Acta Crystallogr. Sect. D Biol. Crystallogr..

[55] Nicholls R. (2017). Ligand fitting with CCP4. Acta Crystallogr. Sect. D.

[56] Chen V.B., Arendall W.B., Headd J.J., Keedy D.A., Immormino R.M., Kapral G.J., Murray L.W., Richardson J.S., Richardson D.C. (2010). MolProbity: All-atom structure validation for macromolecular crystallography. Acta Crystallogr. Sect. D Biol. Crystallogr..

[57] Laskowski R.A., MacArthur M.W., Moss D.S., Thornton J.M. (1993). PROCHECK: A program to check the stereochemical quality of protein structures. J. Appl. Crystallogr..

[58] Lütteke T., von der Lieth C.W. (2004). pdb-care (PDB carbohydrate residue check): A program to support annotation of complex carbohydrate structures in PDB files. BMC Bioinform..

[59] Zheng H., Cooper D.R., Porebski P.J., Shabalin I.G., Handing K.B., Minor W. (2017). CheckMyMetal: A macromolecular metal-binding validation tool. Acta Cryst. D Struct. Biol..

[60] Gore S., Sanz García E., Hendrickx P.M.S., Gutmanas A., Westbrook J.D., Yang H., Feng Z., Baskaran K., Berrisford J.M., Hudson B.P. (2017). Validation of Structures in the Protein Data Bank. Structure.

[61] Krissinel E., Henrick K. (2007). Inference of macromolecular assemblies from crystalline state. J. Mol. Biol..

[62] Laskowski R.A. (2009). PDBsum new things. Nucleic Acids Res..

[63] Kleywegt G.J., Harris M.R., Zou J.Y., Taylor T.C., Wählby A., Jones T.A. (2004). The Uppsala Electron-Density Server. Acta Crystallogr. Sect. D Biol. Crystallogr..

[64] Liebschner D., Afonine P.V., Moriarty N.W., Poon B.K., Sobolev O.V., Terwilliger T.C., Adams P.D. (2017). Polder maps: Improving OMIT maps by excluding bulk solvent. Acta Cryst. D Struct. Biol..

[65] Liu W., Xie Y., Ma J., Luo X., Nie P., Zuo Z., Lahrmann U., Zhao Q., Zheng Y., Zhao Y. (2015). IBS: An illustrator for the presentation and visualization of biological sequences. Bioinformatics.

[66] Fang Q., Shen B. (2010). Optimization of polyethylenimine-mediated transient transfection using response surface methodology design. Electron. J. Biotechnol..

[67] Bollin F., Dechavanne V., Chevalet L. (2011). Design of Experiment in CHO and HEK transient transfection condition optimization. Protein Expr. Purif..

[68] De Los Milagros Bassani Molinas M., Beer C., Hesse F., Wirth M., Wagner R. (2013). Optimizing the transient transfection process of HEK-293 suspension cells for protein production by nucleotide ratio monitoring. Cytotechnology.

[69] Sun S., Wang Q., Zhao F., Chen W., Li Z. (2011). Glycosylation Site Alteration in the Evolution of Influenza A (H1N1) Viruses. PLoS ONE.

[70] Varghese J.N., McKimm-Breschkin J.L., Caldwell J.B., Kortt A.A., Colman P.M. (1992). The structure of the complex between influenza virus neuraminidase and sialic acid, the viral receptor. Proteins.

[71] McAuley J.L., Gilbertson B.P., Trifkovic S., Brown L.E., McKimm-Breschkin J.L. (2019). Influenza Virus Neuraminidase Structure and Functions. Front Microbiol..

[72] Harding M.M. (2001). Geometry of metal-ligand interactions in proteins. Acta Crystallogr. Sect. D Biol. Crystallogr..

[73] Russell R.J., Haire L.F., Stevens D.J., Collins P.J., Lin Y.P., Blackburn G.M., Hay A.J., Gamblin S.J., Skehel J.J. (2006). The structure of H5N1 avian influenza neuraminidase suggests new opportunities for drug design. Nature.

[74] Amaro R.E., Minh D.D.L., Cheng L.S., Lindstrom W.M., Olson A.J., Lin J.-H., Li W.W., McCammon J.A. (2007). Remarkable Loop Flexibility in Avian Influenza N1 and Its Implications for Antiviral Drug Design. J. Am. Chem. Soc..

[75] Wang M., Qi J., Liu Y., Vavricka C.J., Wu Y., Li Q., Gao G.F. (2011). Influenza A virus N5 neuraminidase has an extended 150-cavity. J. Virol..

[76] Smith B.J., Colman P.M., Von Itzstein M., Danylec B., Varghese J.N. (2001). Analysis of inhibitor binding in influenza virus neuraminidase. Protein Sci. A Publ. Protein Soc..

[77] Vavricka C.J., Li Q., Wu Y., Qi J., Wang M., Liu Y., Gao F., Liu J., Feng E., He J. (2011). Structural and functional analysis of laninamivir and its octanoate prodrug reveals group specific mechanisms for influenza NA inhibition. PLoS Pathog..

[78] Kerry P.S., Mohan S., Russell R.J., Bance N., Niikura M., Pinto B.M. (2013). Structural basis for a class of nanomolar influenza A neuraminidase inhibitors. Sci. Rep..

[79] Li Q., Qi J., Zhang W., Vavricka C.J., Shi Y., Wei J., Feng E., Shen J., Chen J., Liu D. (2010). The 2009 pandemic H1N1 neuraminidase N1 lacks the 150-cavity in its active site. Nat. Struct. Mol. Biol..

[80] Shtyrya Y.A., Mochalova L.V., Bovin N.V. (2009). Influenza Virus Neuraminidase: Structure and Function. Acta Nat..

[81] Kim H., Webster R.G., Webby R.J. (2018). Influenza Virus: Dealing with a Drifting and Shifting Pathogen. Viral Immunol..

[82] (2020). Antiviral drugs for influenza for 2020–2021. Med. Lett. Drugs Ther..

[83] Hayden F.G., Sugaya N., Hirotsu N., Lee N., de Jong M.D., Hurt A.C., Ishida T., Sekino H., Yamada K., Portsmouth S. (2018). Baloxavir Marboxil for Uncomplicated Influenza in Adults and Adolescents. N. Engl. J. Med..

[84] Park J.H., Kim B., Antigua K.J.C., Jeong J.H., Kim C.I., Choi W.S., Oh S., Kim C.H., Kim E.G., Choi Y.K. (2021). Baloxavir-oseltamivir combination therapy inhibits the emergence of resistant substitutions in influenza A virus PA gene in a mouse model. Antivir. Res..

[85] Mayer M.P., Bukau B. (2005). Hsp70 chaperones: Cellular functions and molecular mechanism. Cell. Mol. Life Sci..

[86] Östbye H., Gao J., Martinez M.R., Wang H., de Gier J.W., Daniels R. (2020). N-Linked Glycan Sites on the Influenza A Virus Neuraminidase Head Domain Are Required for Efficient Viral Incorporation and Replication. J. Virol..

[87] Bao D., Xue R., Zhang M., Lu C., Ma T., Ren C., Zhang T., Yang J., Teng Q., Li X. (2021). N-Linked Glycosylation Plays an Important Role in Budding of Neuraminidase Protein and Virulence of Influenza Viruses. J. Virol..

[88] Kim P., Jang Y.H., Kwon S.B., Lee C.M., Han G., Seong B.L. (2018). Glycosylation of Hemagglutinin and Neuraminidase of Influenza A Virus as Signature for Ecological Spillover and Adaptation among Influenza Reservoirs. Viruses.

[89] Wu Z.L., Zhou H., Ethen C.M., Reinhold V.N. (2016). Core-6 fucose and the oligomerization of the 1918 pandemic influenza viral neuraminidase. Biochem. Biophys. Res. Commun..

[90] Wu Z.L., Ethen C., Hickey G.E., Jiang W. (2009). Active 1918 pandemic flu viral neuraminidase has distinct N-glycan profile and is resistant to trypsin digestion. Biochem. Biophys. Res. Commun..

[91] Von Grafenstein S., Wallnoefer H.G., Kirchmair J., Fuchs J.E., Huber R.G., Schmidtke M., Sauerbrei A., Rollinger J.M., Liedl K.R. (2015). Interface dynamics explain assembly dependency of influenza neuraminidase catalytic activity. J. Biomol. Struct. Dyn..

[92] Saito T., Taylor G., Webster R.G. (1995). Steps in maturation of influenza A virus neuraminidase. J. Virol..

[93] Gruswitz F., Chaudhary S., Ho J.D., Schlessinger A., Pezeshki B., Ho C.-M., Sali A., Westhoff C.M., Stroud R.M. (2010). Function of human Rh based on structure of RhCG at 2.1 Å. Proc. Natl. Acad. Sci. USA.

[94] Standfuss J., Xie G., Edwards P.C., Burghammer M., Oprian D.D., Schertler G.F.X. (2007). Crystal Structure of a Thermally Stable Rhodopsin Mutant. J. Mol. Biol..

[95] Penmatsa A., Wang K.H., Gouaux E. (2013). X-ray structure of dopamine transporter elucidates antidepressant mechanism. Nature.

[96] Li J., zu Dohna H., Cardona C.J., Miller J., Carpenter T.E. (2011). Emergence and Genetic Variation of Neuraminidase Stalk Deletions in Avian Influenza Viruses. PLoS ONE.

[97] Li Y., Chen S., Zhang X., Fu Q., Zhang Z., Shi S., Zhu Y., Gu M., Peng D., Liu X. (2014). A 20-amino-acid deletion in the neuraminidase stalk and a five-amino-acid deletion in the NS1 protein both contribute to the pathogenicity of H5N1 avian influenza viruses in mallard ducks. PLoS ONE.

