# Supplementary material for: Optimisation of Neuraminidase Expression for Use in Drug Discovery by Using HEK293-6E Cells
Source: Viruses. 2021 Sep 22;13(10):1893. doi: 10.3390/v13101893 (PMC8538103; doi:10.3390/v13101893)
Supplement: Supplementary file 1 [file viruses-13-01893-s001.zip › viruses-1355856-supplementary.pdf]

Supplementary Materials for

Optimisation of Neuraminidase Expression for Use in Drug Discovery by Using HEK293-6E Cells

Ashley C. Campbell[1], John J. Tanner[2,3] and Kurt L. Krause[1,4*]

[1] Department of Biochemistry, University of Otago, 710 Cumberland St., Dunedin 9016, New Zealand; ashley.campbell@otago.ac.nz
[2] Department of Biochemistry, University of Missouri, Columbia, MO, 65211, USA; tannerjj@missouri.edu
[3] Department of Chemistry, University of Missouri, Columbia, MO, 65211, USA
[4] Maurice Wilkins Centre for Molecular Biodiscovery, Auckland, 1010, New Zealand

* Correspondence: kurt.krause@otago.ac.nz;

This PDF file includes:

Figures S1 to S7

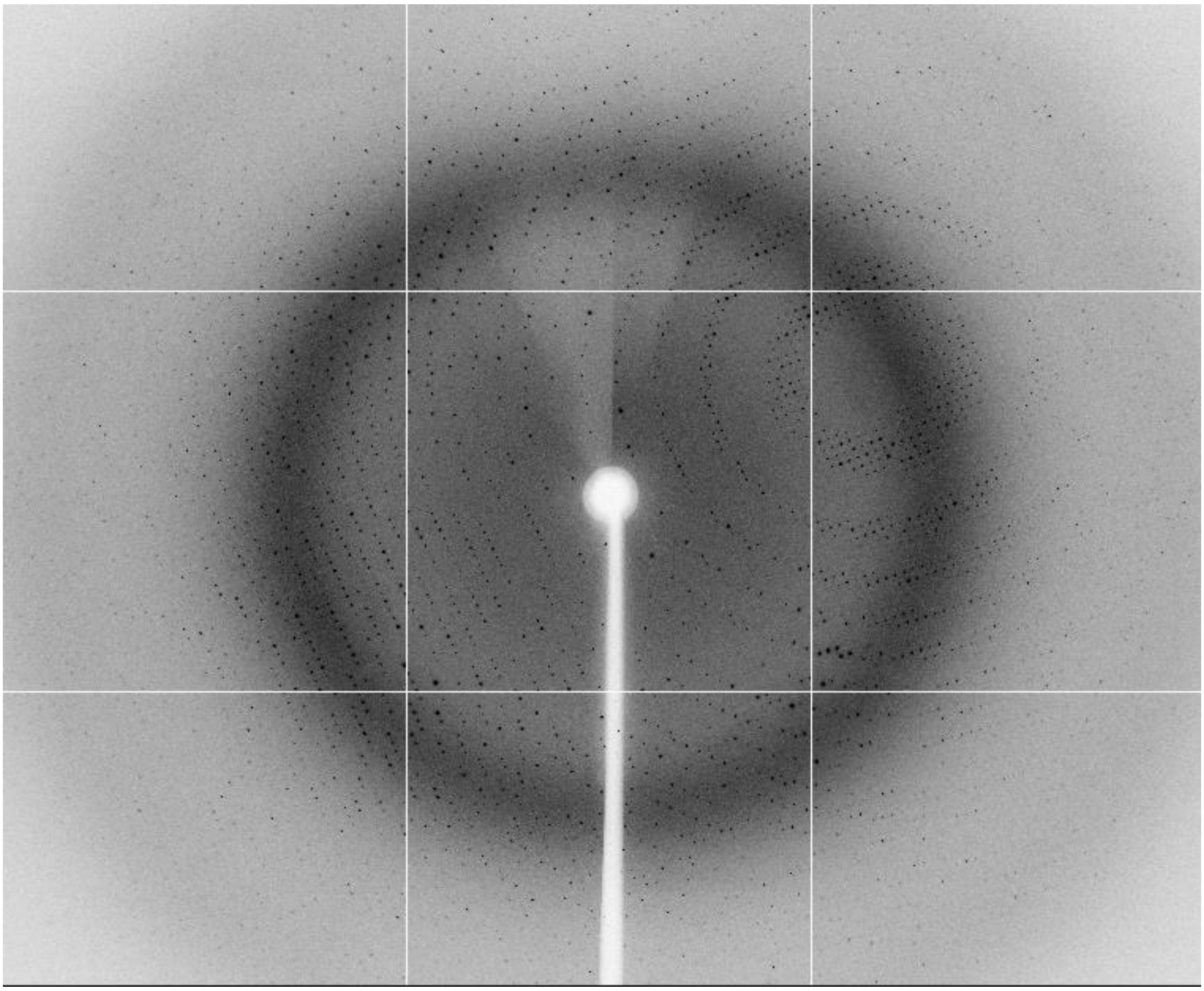

Figure S1 – Enlarged X-ray diffraction image from an H1N1 NA crystal. This image was collected at the MX2 beamline at the Australian Synchrotron. Maximum resolution at the edge of the detector is 2.04 Å, beam energy 12664.93 eV, detector distance 300 mm, exposure time 1 s.

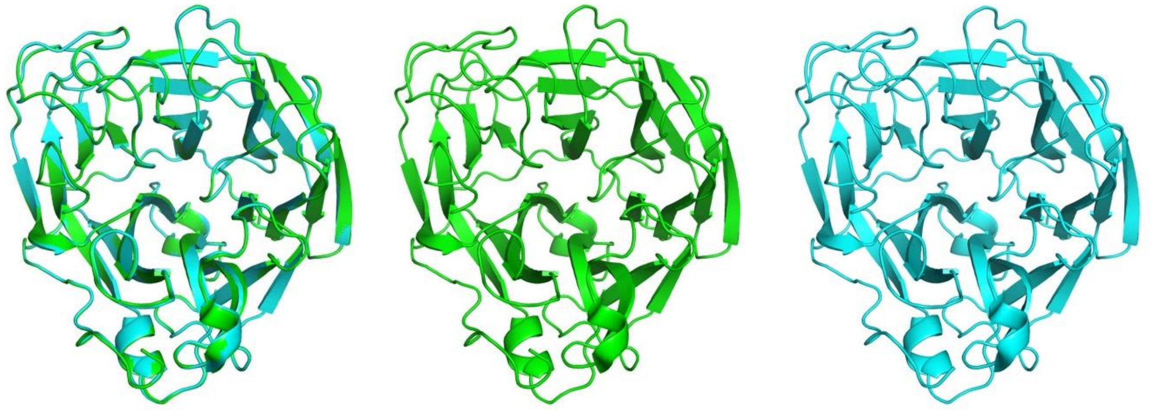

Figure S2 – Side-by-side comparison of HEK NA and 3BEQ. Structural alignment of NA monomers from influenza A/BrevigMission/1/1918(H1N1) using different expression systems. Overlay of both structures showing close alignment, Lime green HEK NA, Cyan 3BEQ. This superposition was performed in PyMOL, using the align command, which performs a sequence alignment, followed by a structural superposition. Water, metal ions and ligands were excluded from the alignment. Chain A from each structure was used for this alignment.

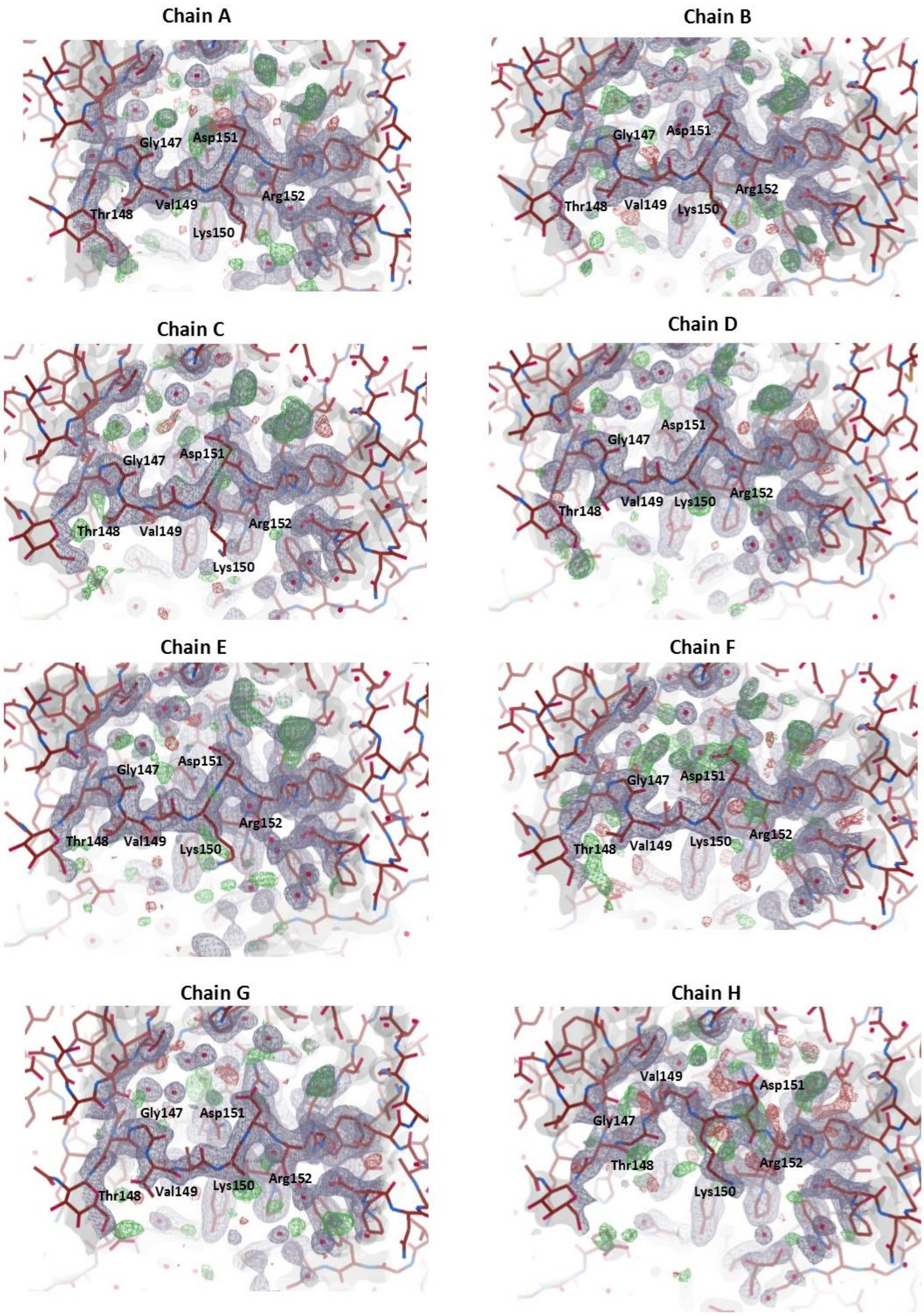

Figure S3 - 150-cavity of the 8 chains in HEK NA. Inspection indicated that the cavity structure is predominantly open. Electron density for chains A-H is shown near the 150-cavity superposed onto the refined NA structure. These images were made in Coot, with the 2Fo-Fc map (grey) contoured to 1σ and the Fo-Fc map (positive-green and negative-red) contoured to 3σ.

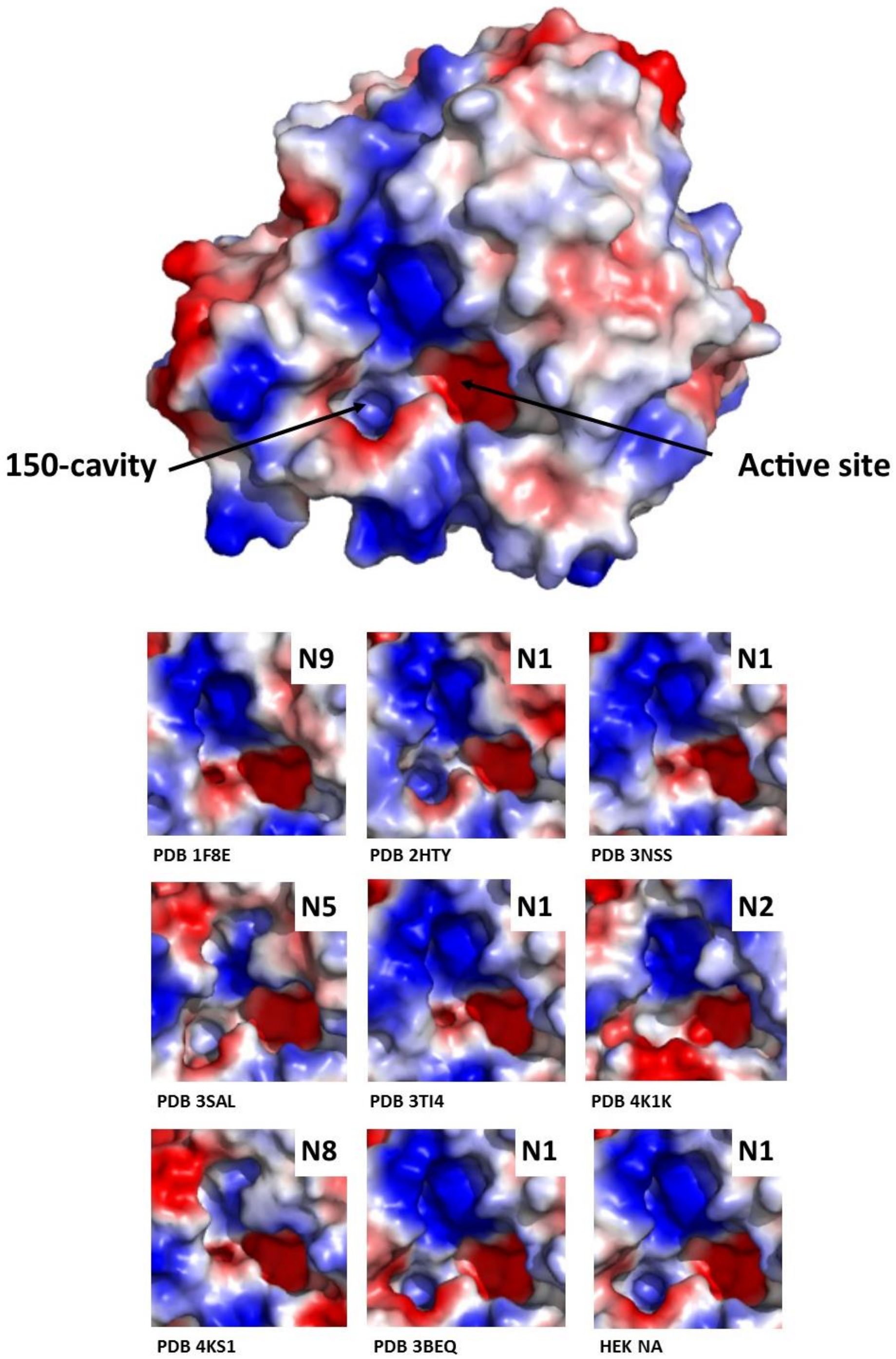

Figure S4 - Active site and 150-cavity of selected NA structures. The molecular surfaces of selected NA monomers from the PDB (see Table 3) are displayed near their 150-cavity. Structures were aligned in PyMOL using the Align command as described earlier. Electrostatic surface representation is displayed as a charge-smoothed potential with positive regions in blue and more negative regions shown in red.

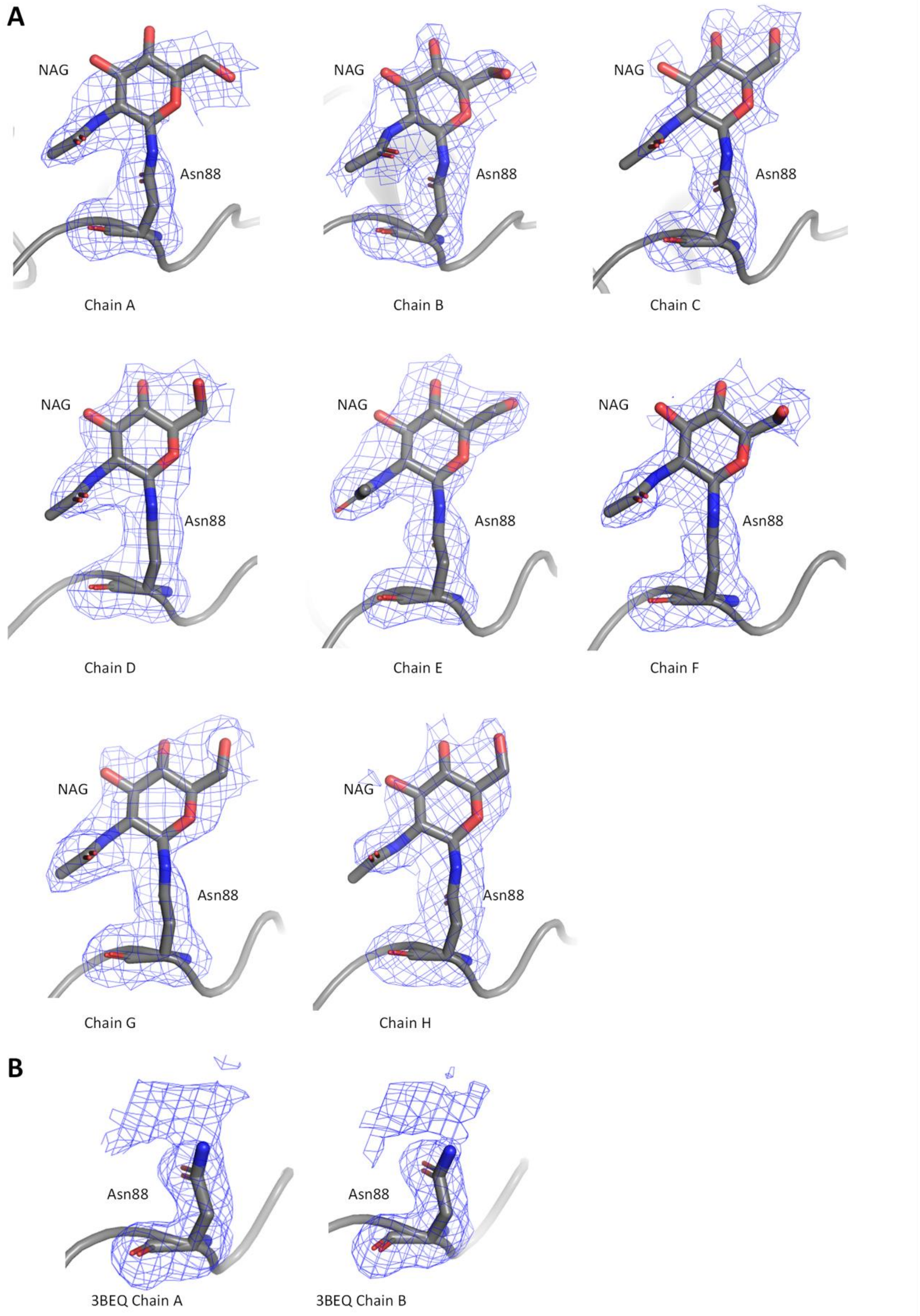

Figure S5 – NA glycosylations at Asn88. A) Electron density for chains A-H is shown near Asn88 superposed onto the refined NA structure. These images were made in PyMOL, with polder maps (blue mesh) contoured to 4σ. B) For comparison the electron density for Asn88 in chains A-B from 3BEQ the insect cell H1N1 NA structure.

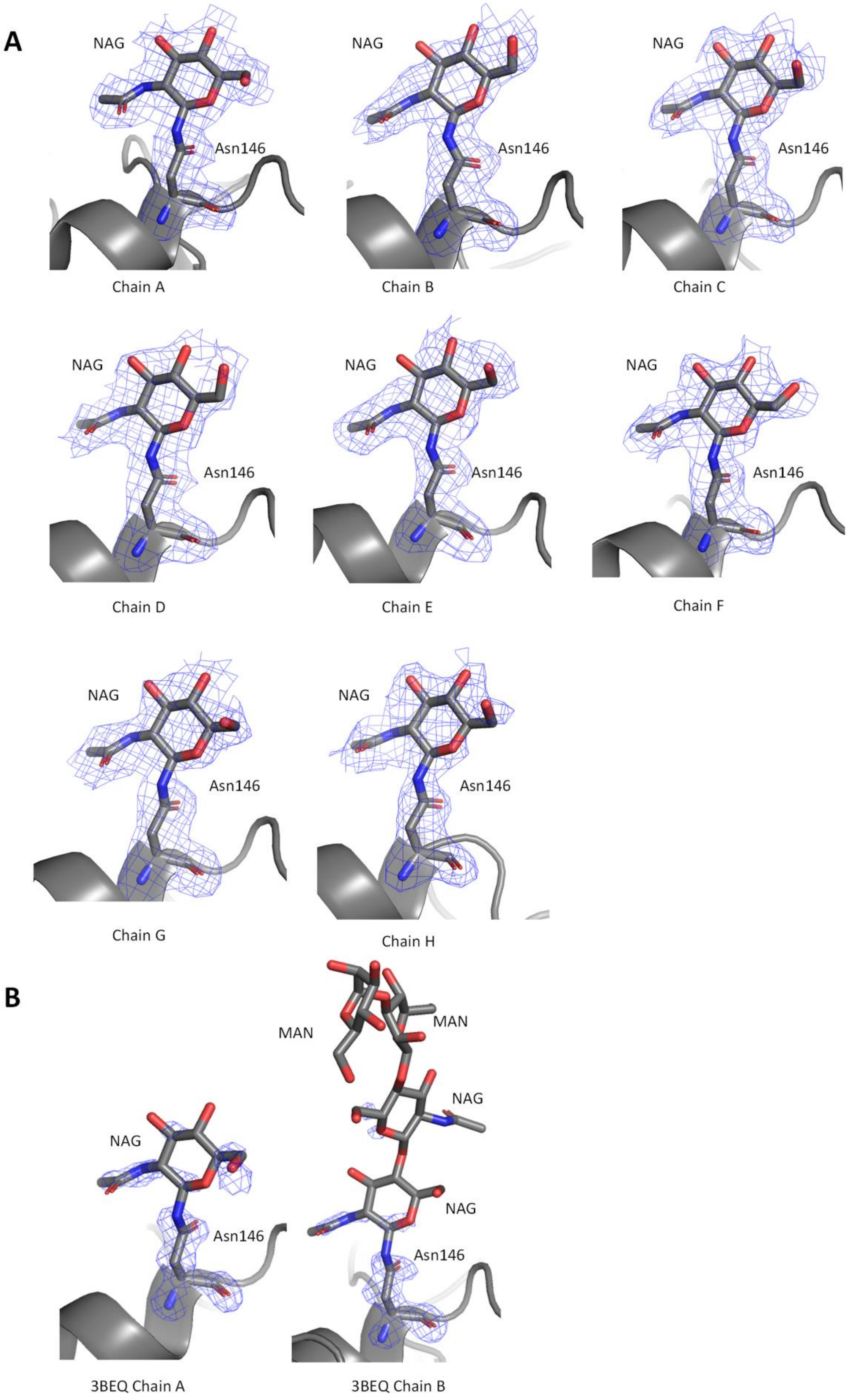

Figure S6 –NA glycosylations at Asn146. A) Electron density for chains A-H is shown near Asn146 superposed onto the refined NA structure. These images were made in PyMOL, with polder maps (blue mesh) contoured to 4σ. B) For comparison the electron density for Asn146 in chains A-B from 3BEQ the insect cell H1N1 NA structure.

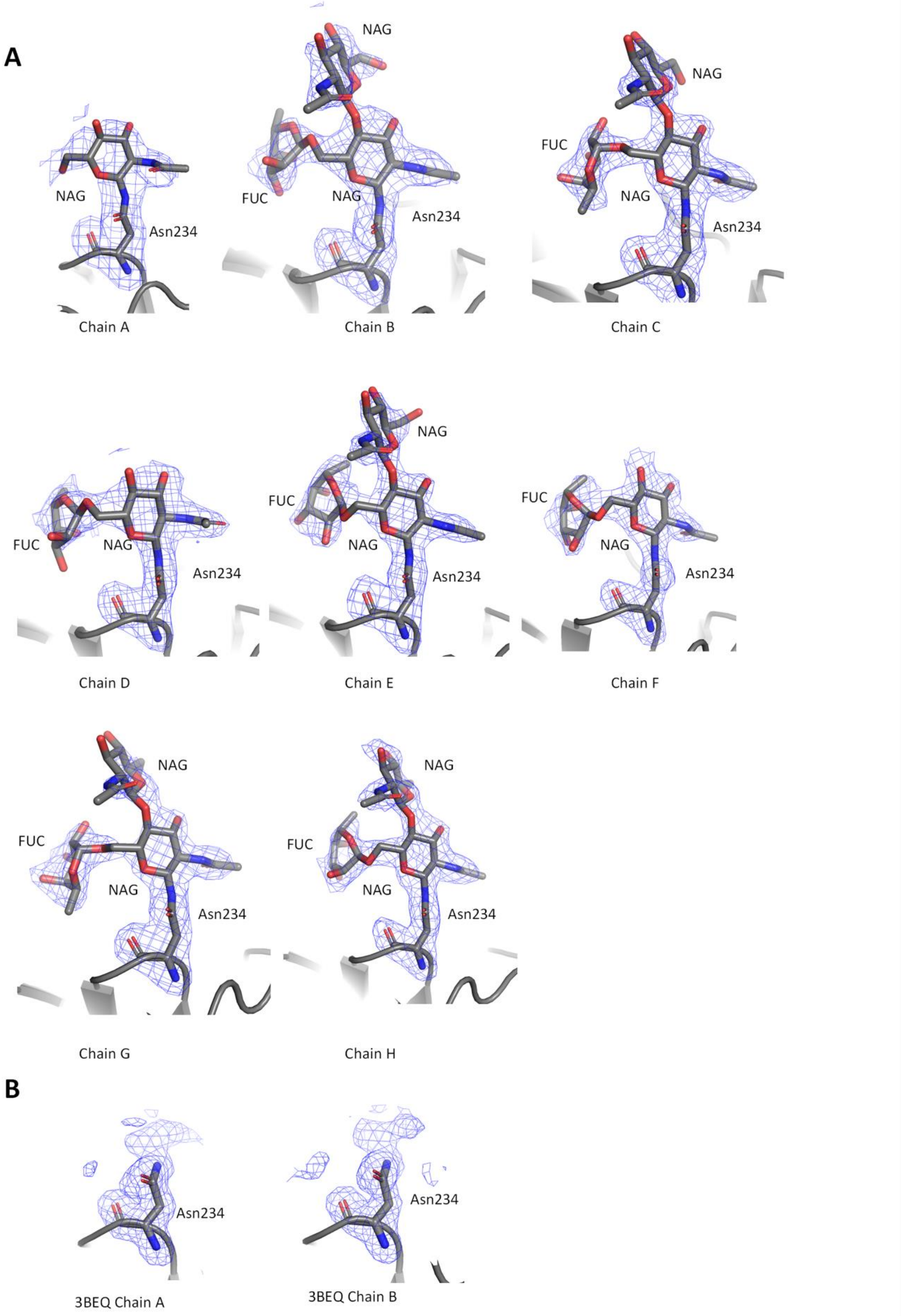

Figure S7 - NA glycosylations at Asn234. A) Electron density for chains A-H is shown near Asn234 superposed onto the refined NA structure. These images were made in PyMOL, with polder maps (blue mesh) contoured to 4σ. B) For comparison the electron density for Asn234 in chains A-B from 3BEQ the insect cell H1N1 NA structure.
